# Transfer cells in *Horneophyton lignieri* illuminate the origin of vascular tissues in land plants

**DOI:** 10.1111/nph.70850

**Published:** 2025-12-17

**Authors:** Paul Kenrick, Emma J. Long

**Affiliations:** ^1^ Natural History Museum Science Group Cromwell Road London SW7 5BD UK; ^2^ Centre for Ecology & Conservation University of Exeter Penryn Campus Penryn TR10 9FE UK

**Keywords:** confocal laser scanning microscopy, Devonian, *Horneophyton lignieri*, phloem, Rhynie Chert, transfer cells, vascular system, xylem

## Abstract

- Recent fossil discoveries and advances in plant phylogeny have renewed debate about the most recent common ancestor (MRCA) of land plants and the evolution of its fundamental organs and tissues. We re‐investigate the vascular system of *Horneophyton lignieri*, an exceptionally preserved Rhynie Chert fossil central to understanding early plant evolution.
- Using confocal laser scanning microscopy combined with 3D modelling, we achieved higher resolution and precision in reconstructing cell morphology than earlier studies that relied on white light microscopy.
- We show that the vascular system of *H. lignieri* lacks distinct xylem and phloem tissues, contrary to prior assumptions. Instead, tissues with transfer cell‐like structures are prominent, and both cell type and cell wall development vary with position in the plant.
- These findings indicate that the ancestral vascular system of land plants likely consisted of a single type of conducting cell capable of both solute transport and water conduction. Our results show that *H. lignieri* is not a tracheophyte, supporting emerging models of a morphologically and cellularly complex MRCA for land plants.

## Introduction

The evolution of the vascular system over 400 million years ago (Ma) marked a transformative milestone in plant evolution. This development allowed plants to substantially increase in size and complexity, setting the stage for the emergence of more diverse and productive terrestrial ecosystems (Leslie *et al*., 2021; Clark *et al*., 2023). Composed mainly of xylem and phloem, the vascular system facilitates the internal transport of fluids, metabolic products, and small signalling molecules, while in many species it also plays crucial biomechanical roles (Lucas *et al*., 2013). It is the defining characteristic of vascular plants, though conducting tissues are also found in some bryophytes, raising the possibility of a common evolutionary origin of these systems in land plants. Bryophyte conducting tissues share significant similarities with the tracheids and sieve elements of xylem and phloem (Hébant, 1977; Scheirer, 1980), though notable differences raise questions regarding their homologies (Ligrone *et al*., 2000, 2012; Renzaglia *et al*., 2007). Recently, molecular developmental research has brought a fresh perspective, suggesting that a complex, shared regulatory framework, based on transcription factors, governs the development of water‐conducting tissues in all plants (Xu *et al*., 2014; Ohtani *et al*., 2016; Bowles *et al*., 2022). Additionally, common molecular mechanisms are observed in the regulation of tracheid and sieve element development within vascular plants (Ohtani *et al*., 2016; Heo *et al*., 2017). These findings, along with new insights into plant phylogeny (Puttick *et al*., 2018) and recent discoveries in the early fossil record (Edwards *et al*., 2021a), have reinvigorated debates about the nature of ancestral land plants and the evolution of their conducting systems (Kenrick, 2018; Donoghue *et al*., 2021; Harris *et al*., 2022; Woudenberg *et al*., 2022). Here, we re‐investigate the vascular system of *Horneophyton lignieri*, an exceptionally well‐preserved fossil that has been central to shaping our understanding of early plant evolution.

**Fig. 1:**
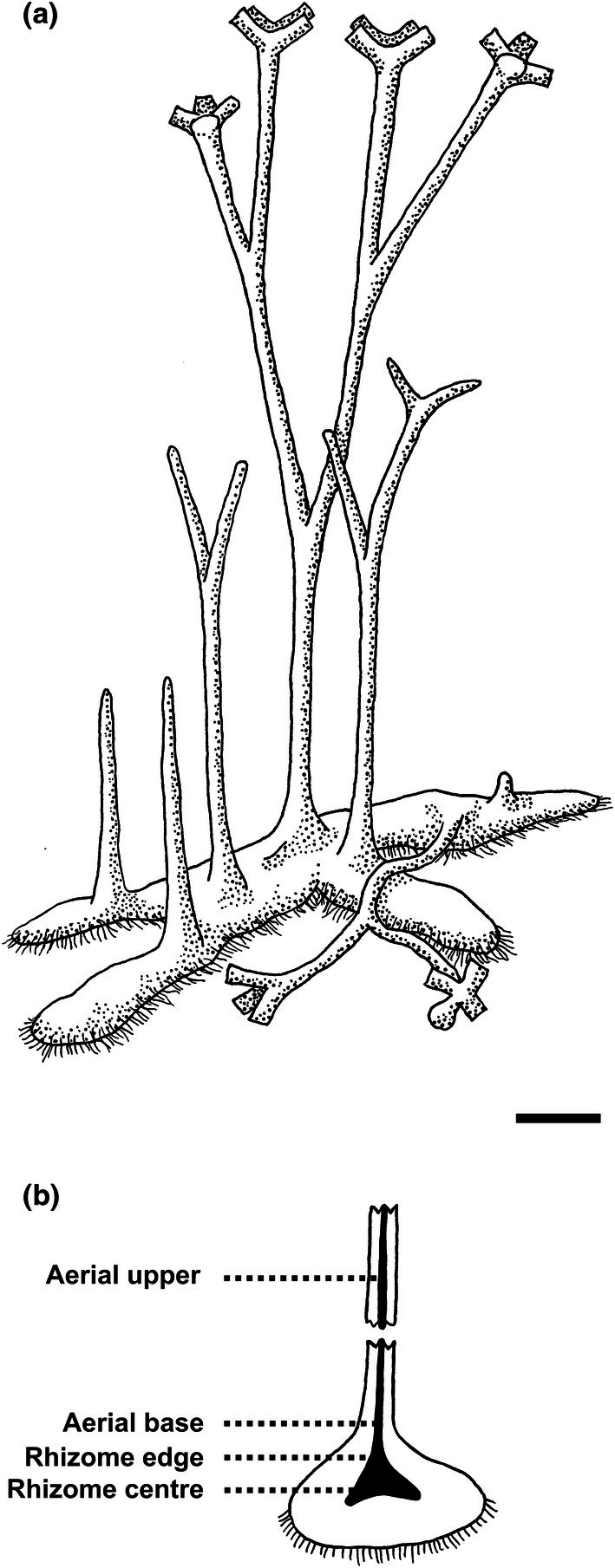
*Horneophyton lignieri* habit and sampling of vascular tissues. (a) Reconstruction: Narrow, ephemeral aerial axes developed from the upper surface of a large, prostrate, rhizoid‐bearing rhizome. When mature, aerial axes bore branched cylindrical sporangia at their apices. Bar, 5 mm. (b) Indicative positions of sampled vascular tissues: Rhizome centre (NHMUK PI In 24697, NHMUK SC 3137), Rhizome edge (NHMUK PI In 24697), Aerial axis base (NHMUK PI In 24697, NHMUK SC 3137), Aerial axis upper (NHMUK OC 1938, transverse sections; NHMUK PI In 24697, NHMUK SC 3137, longitudinal sections).

*Horneophyton lignieri* is one of seven land plants from the renowned 408‐million‐year‐old Rhynie Chert, the oldest well‐preserved terrestrial ecosystem yet found in the geological record (Edwards *et al*., 2018). It lived in a geothermal wetland where organisms were permineralised in silica, preserving them in exceptional ways. *H. lignieri* was a diminutive, leafless plant, and probably the smallest species in the Rhynie flora, standing < 20 cm tall (Fig. 1a). It grew from a prostrate rhizome, which developed upright, bifurcating axes (Kidston & Lang, 1920, 1921; Eggert, 1974). Spore‐bearing organs were located terminally on the upright axes, forming branched cylinders (Bhutta, 1973; Eggert, 1974; El‐Saadawy & Lacey, 1979; Remy & Remy, 1980). The vascular system was originally described as a stele comprising a ring of phloem encircling a solid xylem core composed of tracheids (Kidston & Lang, 1920). Tracheids were identified by narrow wall thickenings, which appeared as irregularly connected rings or spirals. Evidence for phloem was more circumstantial: the cells encircling the xylem were thin‐walled, uniformly narrow, and elongate, suggesting that they were sieve elements. *H. lignieri* was therefore considered a vascular plant, albeit a much simpler one than any living species.

Subsequent researchers have largely accepted this view (e.g. Edwards, 2004; Taylor *et al*., 2009; Hao & Xue, 2013; Kerp, 2018), but inconsistencies remain. Unlike other Rhynie plants, there is no vascular system in the rhizome (Edwards, 2004; Kerp, 2018), and tracheid thickenings are absent from some xylem cells (Kidston & Lang, 1920). Eggert (1974) noted that wall thickenings in the tracheids are obscure, and he drew attention to the similarity between phloem cells and collenchyma, a support tissue rather than one involved in conduction. Kenrick & Crane (1997a) further argued that well‐defined wall thickenings characteristic of tracheids are absent, emphasising that the structures observed by previous authors are faint and ephemeral, while Kerp *et al*. (2004) observed cells with uniformly thick walls in xylem‐like tissues of the gametophyte. More recently, Cascales‐Miñana *et al*. (2019) reaffirmed the presence of tracheid‐like wall thickenings. However, the discovery of diverse new types of conducting cells in the early fossil record preserved in charcoal further complicates the narrative (Edwards *et al*., 2003, 2021a,b, 2022). These cells exhibit features superficially resembling tracheid thickenings, as well as characteristics akin to food‐conducting cells in bryophytes and transfer cells (Edwards *et al*., 2003, 2021a,b, 2022). In the light of these inconsistencies and new findings, the vascular system of *H. lignieri* merits re‐evaluation.

Previous studies of *H. lignieri* have exclusively employed white light microscopy, which has inherent limitations when imaging silicified fossils. Out‐of‐focus light causes image blurring, reducing clarity in volumetric data (Shi *et al*., 2013), and optical artefacts may arise through merging of features on distinct focal planes (Long *et al*., 2024a). These issues become increasingly problematic at higher magnifications when interpreting cellular and subcellular structures, making observation of individual focal planes essential. Confocal laser scanning microscopy (CLSM), combined with 3D modelling, provides a solution to these challenges, offering greater clarity in rendering complex morphologies (e.g. Ball *et al*., 2017; Kamanli *et al*., 2017). CLSM relies on autofluorescence and has been successfully applied to diverse Rhynie fossils, including fungi (Strullu‐Derrien *et al*., 2016, 2018, 2023b), cyanobacteria (Strullu‐Derrien *et al*., 2023a), testate amoebae (Strullu‐Derrien *et al*., 2019a), green algae (Strullu‐Derrien *et al*., 2021) and arthropods (Edgecombe *et al*., 2020; Long *et al*., 2024a,b).

In this study, we apply CLSM and 3D modelling to the vascular system of a Rhynie Chert plant for the first time. This approach allows us to re‐evaluate the interpretation of tracheids in *H. lignieri* and to examine how cellular characteristics change across different parts of the plant. Comparisons are drawn with novel conducting cells preserved in fossil charcoal of a broadly comparable age.

## Materials and Methods

### Geological site, environment and preparations

The Rhynie Chert geological site, located near the village of Rhynie in Scotland (57°20′12″N, 2°50′29″W), lies *c*. 50 km north‐west of Aberdeen. Detailed information about its geological setting, age and palaeoenvironment is available in a series of papers arising from a discussion meeting held at The Royal Society, London (Edwards *et al*., 2018) and in two recent reviews (Strullu‐Derrien *et al*., 2019b; Garwood *et al*., 2020). Radiometric dating estimates the age of chert deposition to be between 407.6 ± 2.2 Ma (Mark *et al*., 2013) and 411.5 ± 1.3 Ma (Parry *et al*., 2013), with differences attributed to methodological variations, potential sources of error, and underlying geological assumptions (Trewin & Kerp, 2017; Edwards *et al*., 2018). These radiometric ages align broadly with the biostratigraphic interval indicated by the spore assemblage biozone (late Pragian to possibly earliest Emsian; Wellman, 2006) and the latest numerical ages for Devonian Period stage boundaries (Cohen *et al*., 2013, updated).

The Rhynie Chert environment is interpreted as a low‐energy alluvial plain where plants thrived on sandy substrates or siliceous sinter surfaces near a river system with ephemeral ponds and small lakes (Trewin, 1994; Rice *et al*., 2002). Silica‐enriched water from a local hydrothermal source rapidly petrified the plants, entombing them in bands of hard chert, retaining much of the original ecological context. Silicification occurred at low temperatures (< 50°C), preserving exceptional cellular detail (Trewin & Fayers, 2016; Channing, 2018; Parnell *et al*., 2023). From the preserved spatial relationships of the fossils, we can infer that these small plants grew in dense carpets of vegetation, often in monotypic stands. Within chert bands, *Horneophyton lignieri* (Kidston & Lang) Barghoorn & Darrah commonly co‐occurs with *Rhynia gwynne‐vaughanii* Kidston & Lang, the latter preferring sandy substrates and *H. lignieri* preferring sinter surfaces (Powell *et al*., 2000).

Historical thin sections of Rhynie Chert prepared by Walter Hemingway (1859–1947; Stevenson, 2016) were examined from collections at the Natural History Museum, London (NHMUK). Of 24 sections containing *Horneophyton lignieri* (≡ *Hornea lignieri*, Barghoorn & Darrah, 1938), three were selected for detailed imaging based on cellular preservation, position within the plant, and section orientation. Slides contained multiple axes of *H. lignieri* and are catalogued under NHMUK PI In 24697, NHMUK OC 1938 and NHMUK SC 3137. Details of taxonomic verification are given in Supporting Information Methods S1.

### White light microscopy

Thin sections were imaged using white light microscopy (WLM) at the Digitisation and Microscopy Laboratory of the Imaging and Analysis Centre at the Natural History Museum, London, with an Olympus BX63 microscope (Olympus, Tokyo, Japan) fitted with an Olympus DP73 camera. Z‐stacks were acquired under brightfield illumination using Olympus cellSens Dimension software (v.1.15). Overview and close‐up images were taken with ×4 and ×40 objective lenses, respectively. Stacks were rendered into extended‐depth‐of‐focus images using rendering method C in Helicon Focus Pro (v.6.3.0).

### Confocal laser scanning microscopy

Confocal image stacks were obtained at the Imaging and Analysis Centre at the Natural History Museum, London, using a Nikon A1‐Si confocal laser scanning microscope mounted on a Nikon Eclipse E upright microscope. Autofluorescence was excited with a 640 nm laser (Coherent Inc., USA) and detected at 675–725 nm. Signal quality was enhanced using line averaging (×4 or ×16), and the pinhole aperture was adjusted to between *c*. 30 and 40 μm. Most imaging used a ×40 Plan Fluor Oil objective, with additional imaging using a Plan Fluor ×20 Air objective. Larger scans were created by stitching overlapping z‐stack tiles using Nikon Nis Elements software (v.4.60). Detailed scan parameters are provided in Table S1. Image stacks are deposited at morphosource.org.

### Post‐imaging 3D visualisation and modelling

Confocal data were visualised using Avizo 3D (v.2021.2; Thermo Fisher Scientific, Waltham, MA, USA) on a Dell workstation (Intel® Xeon® Gold 5218R 2.10 GHz; 256 GB RAM). Avizo tools and modules are capitalised and italicised herein. Image stacks (typically 65–250 slices) were inspected using the *Ortho Slice* viewer, adjusting *Colormap* ranges to emphasise mid‐range grayscale values. To facilitate comparison with white‐light microscopy, a *grayScaleInverted Colormap* was applied (high fluorescence = dark; low fluorescence = light). Volumes were created using *Volume Rendering* with *Standard Rendering*, *Edge 2D Shade Effects*, *Diffuse Lighting*, and a *grayScale Colormap*. *Opacity* transfer functions were adjusted to suppress background signal (e.g. anomalous fluorescence in the surrounding chert matrix) and enhance internal anatomy. Virtual dissections were performed using the *Clip* tool. Images were exported using the *Camera* tool as RGB‐alpha *.*tif* files.

### Measurements and statistical analysis

Cell and cell wall dimensions were measured from single orthogonal confocal sections using Fiji (Schindelin *et al*., 2012). Measurements were imported into Microsoft Excel for graphical and statistical analysis. Quantile–quantile plots confirmed data normality. Student's *t*‐tests (two‐tailed; two‐sample unequal variance; *P*‐value < 0.05) were used to compare means between paired groups. Summary statistics are provided in Tables S2–S5.

## Results

The analysis presented here focuses on cells that belong to the vascular system of *H. lignieri* based on their positions and structures. When observed in the aerial axes, these cells were formerly identified as xylem and phloem. While previous works agree that the rhizome lacked vascular tissues, it contains conspicuous clusters of cells with relatively thick, brown‐coloured walls. These cells are located toward the centre of the rhizome and beneath the points from which aerial axes developed. Their function is not understood, but they are known to connect with the vascular tissues of the aerial axes. We analysed these tissues collectively but refer to them using the more neutral term ‘conducting cells’.

### Morphology of conducting cells and associated tissues

**Fig. 2:**
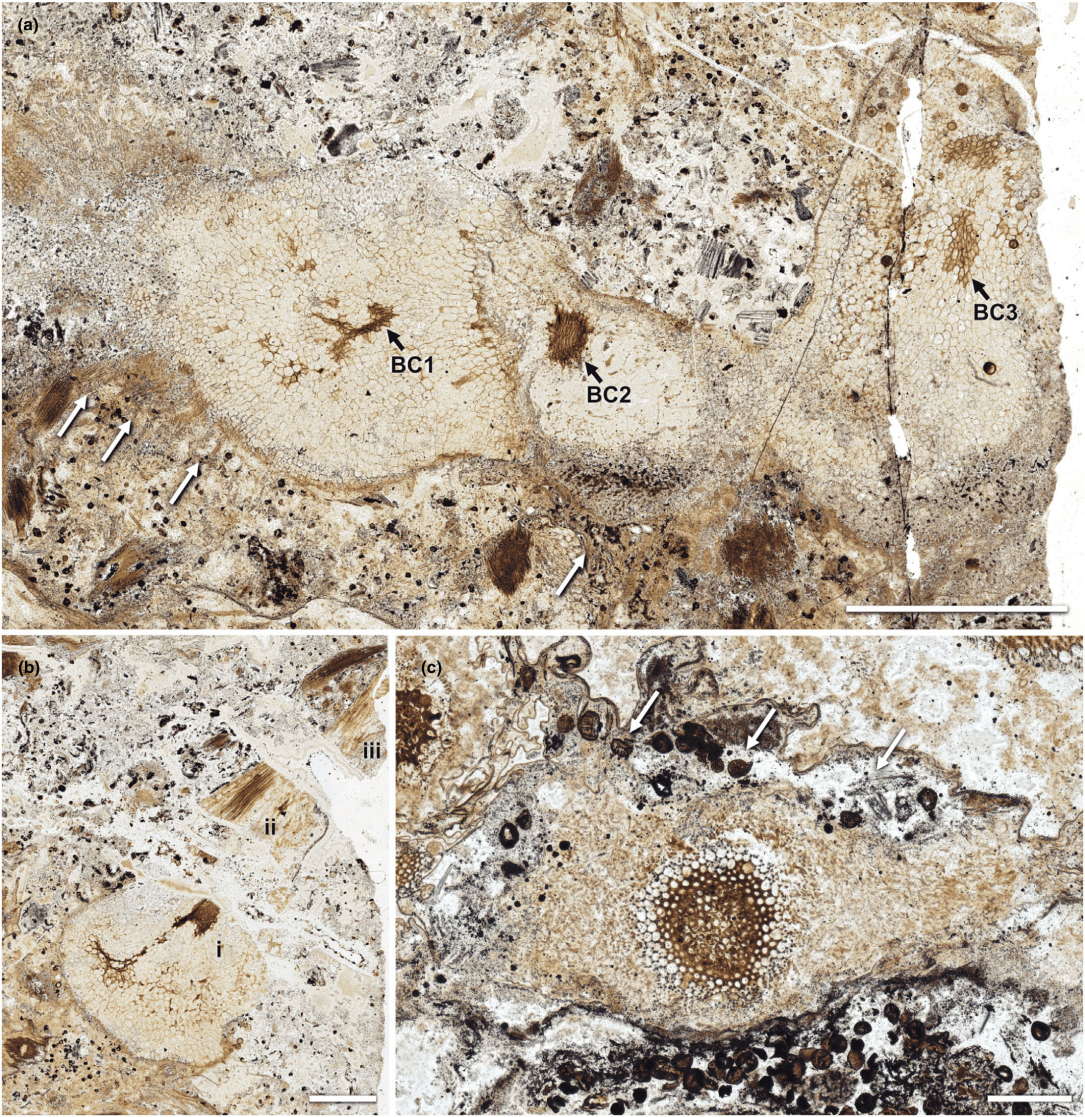
White light images of rhizomes and aerial axes of *Horneophyton lignieri*, illustrating the broader anatomical context for features examined in detail in subsequent figures. (a) Section through rhizome showing three loci of brown‐walled rhizome centre cells (BC). BC3 is shown at higher magnification in subsequent figures (NHMUK PI In 24697). White arrows point to areas with rhizoids. (b) Vertical section through rhizome (i) and base of aerial axis (ii, iii). The section has fractured into three separate pieces (i, ii, iii). Brown‐walled rhizome edge cells (i) depicted at higher magnification in subsequent figures (NHMUK PI In 24697). (c) Transverse section of aerial axis showing cellular preservation of central conducting tissues and collapse of peripheral tissues with ingress of spores (arrows) (NHMUK OC 1938). Bars: (a) 2 mm; (b) 1 mm; (c) 0.5 mm.

**Fig. 3:**
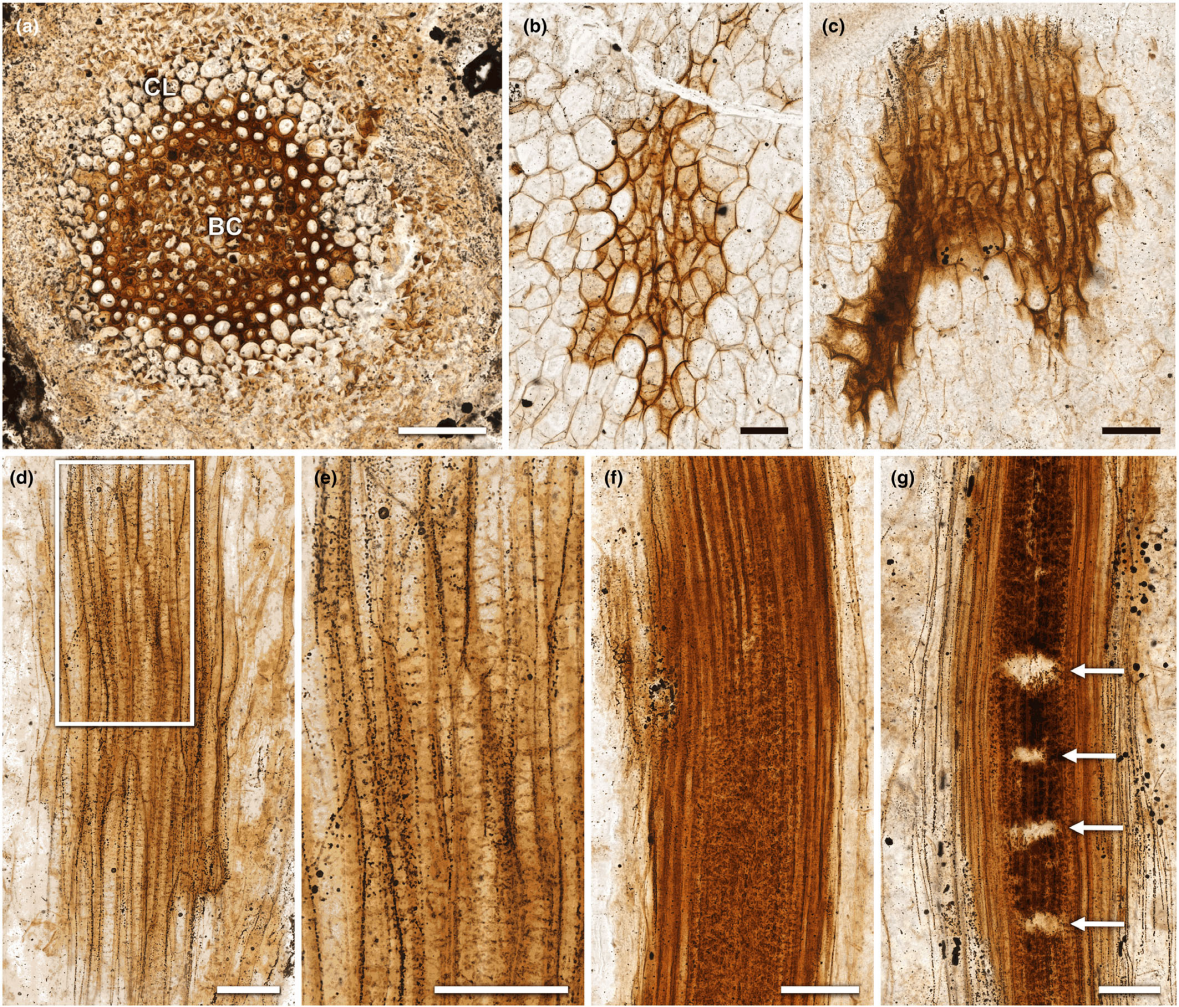
White light images of conducting cells and associated tissues in *Horneophyton lignieri*. Thick‐walled conducting cells, corresponding to the xylem of Kidston & Lang (1920), are shown in panels (a, c–g), in both rhizomes and aerial axes. (a) Transverse section of conducting strand of aerial axis. Brown, thick‐walled cells (BC) with an outer halo of pale, thin‐walled collenchyma‐like cells (CL) (NHMUK OC 1938). (b) Oblique section of rhizome centre conducting cells abutting thin‐walled cortical cells (NHMUK PI In 24697). (c) Oblique section of rhizome edge conducting cells (NHMUK PI In 24697). (d) Longitudinal section at base of aerial axis (NHMUK PI In 24697). (e) Detail from inset in (d), showing elongate cells with faint, transverse striations resembling tracheid thickenings. (f) Longitudinal section higher in aerial axis (NHMUK PI In 24697). (g) Longitudinal section higher in aerial axis showing distinctive transverse breaks in inner zone (arrows) (NHMUK SC 3137). Bars, 100 μm.

Observations were made on conducting cells at several levels within the plant, including the rhizome (rhizome centre), the transitional zone between rhizome and aerial axes (rhizome edge), and more distally within aerial axes (Fig. 1b). Typically, these cells have relatively thick walls and are readily identified by their dark brown colour (Figs 2, 3), reflecting a high organic content. The rhizome is a tuberous, lobed, prostrate organ from which much narrower aerial axes arose, and is often compared with, or termed, a corm. Tissues of the rhizome are invariably better preserved than those of the aerial axes. Cellular preservation often encompasses the full range of tissues, including conducting cells, cortical parenchyma, epidermis, and rhizoids (Figs 2a,b, S1a). The aerial axes are narrow, vertically elongate and branch dichotomously. Typically, only conducting tissues remain (Figs 2c, 3a,d–g, S1b).

#### Conducting cells and neighbouring cortical cells of the rhizome centre

Observations were made on three independent rhizomes located on slides NHMUK PI In 24697 and NHMUK SC 3137 (Figs 2a,b, S1a). One lobed rhizome on slide NHMUK PI In 24697 is > 10 mm long and has three distinct and separate centres of brown‐walled conducting cells (Fig. 2a). These are surrounded by thin‐walled cortical cells, which constitute the bulk of the tissue. Towards the periphery, some cells are smaller and appear to be organised into short rows orthogonal to the surface, giving the appearance of an ill‐defined outer cortex. Rhizoids are also visible.

**Fig. 4:**
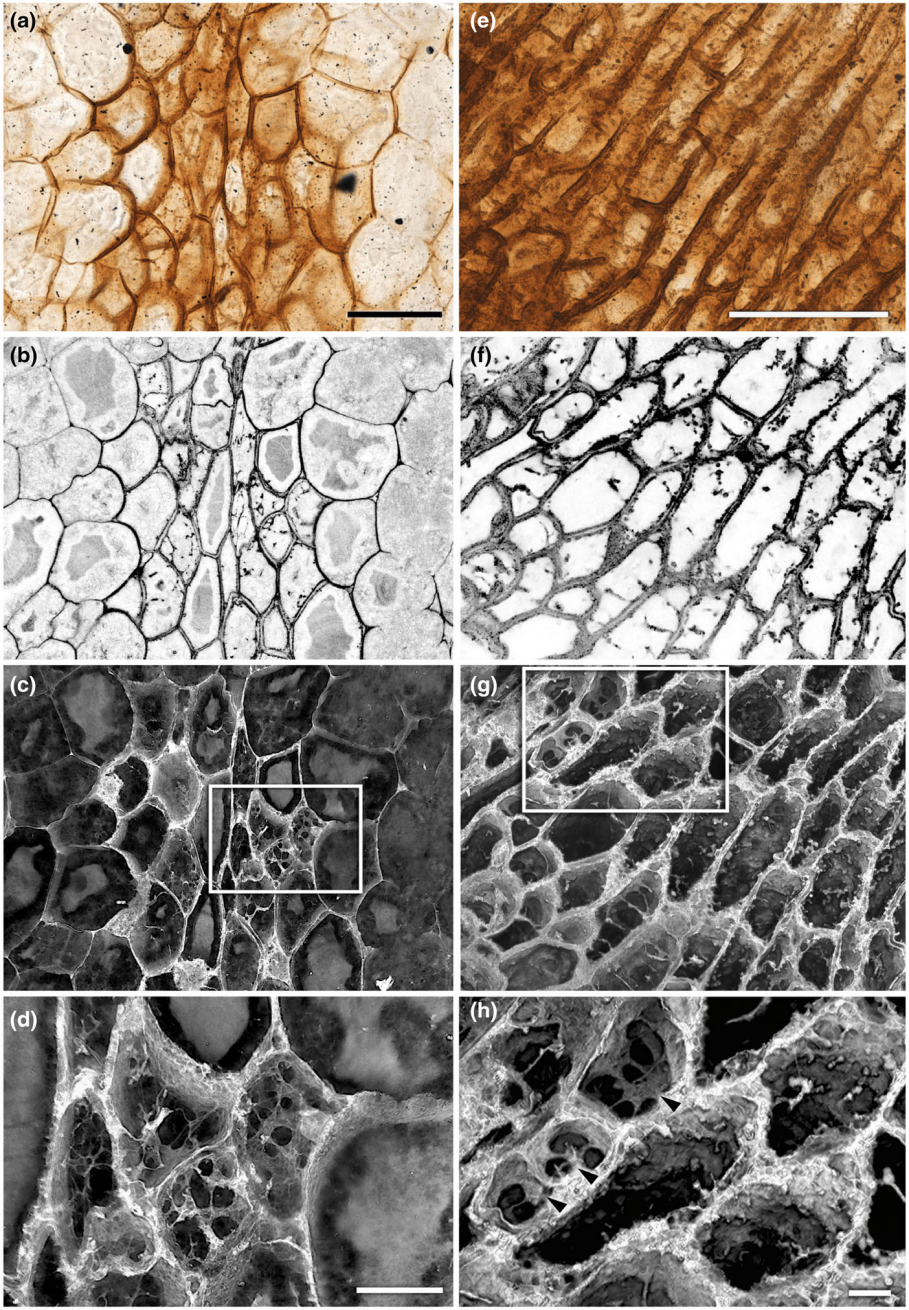
Obliquely oriented transverse section of conducting cells (central, with darker walls) and adjacent cortical cells (larger, with thinner walls) in the rhizome of *Horneophyton lignieri*, imaged using white light microscopy and confocal laser scanning microscopy (NHMUK PI In 24697). (a–c) Rhizome centre: same field of view. (a) White light image. (b) Single confocal optical section revealing the tripartite walls of the conducting cells and strands of material within their lumina. (c) Volume rendering of the confocal image stack providing a 3D view of the same region. (d) Detail from inset in (c), showing a labyrinth of threads and sheets pervading the lumina of conducting cells. (e–f) Rhizome edge. (e) White light image. (f, g) Same field of view. (f) Single confocal optical section showing the tripartite walls of the conducting cells and the presence of lumen strands. (g) Volume rendering. (h) Detail from inset in (g), showing a labyrinthine meshwork within the lumina of conducting cells (arrows) at the periphery. Bars: (a) 100 μm (b, c same scale); (d) 30 μm; (e) 100 μm (f, g same scale); (h) 10 μm.

**Fig. 5:**
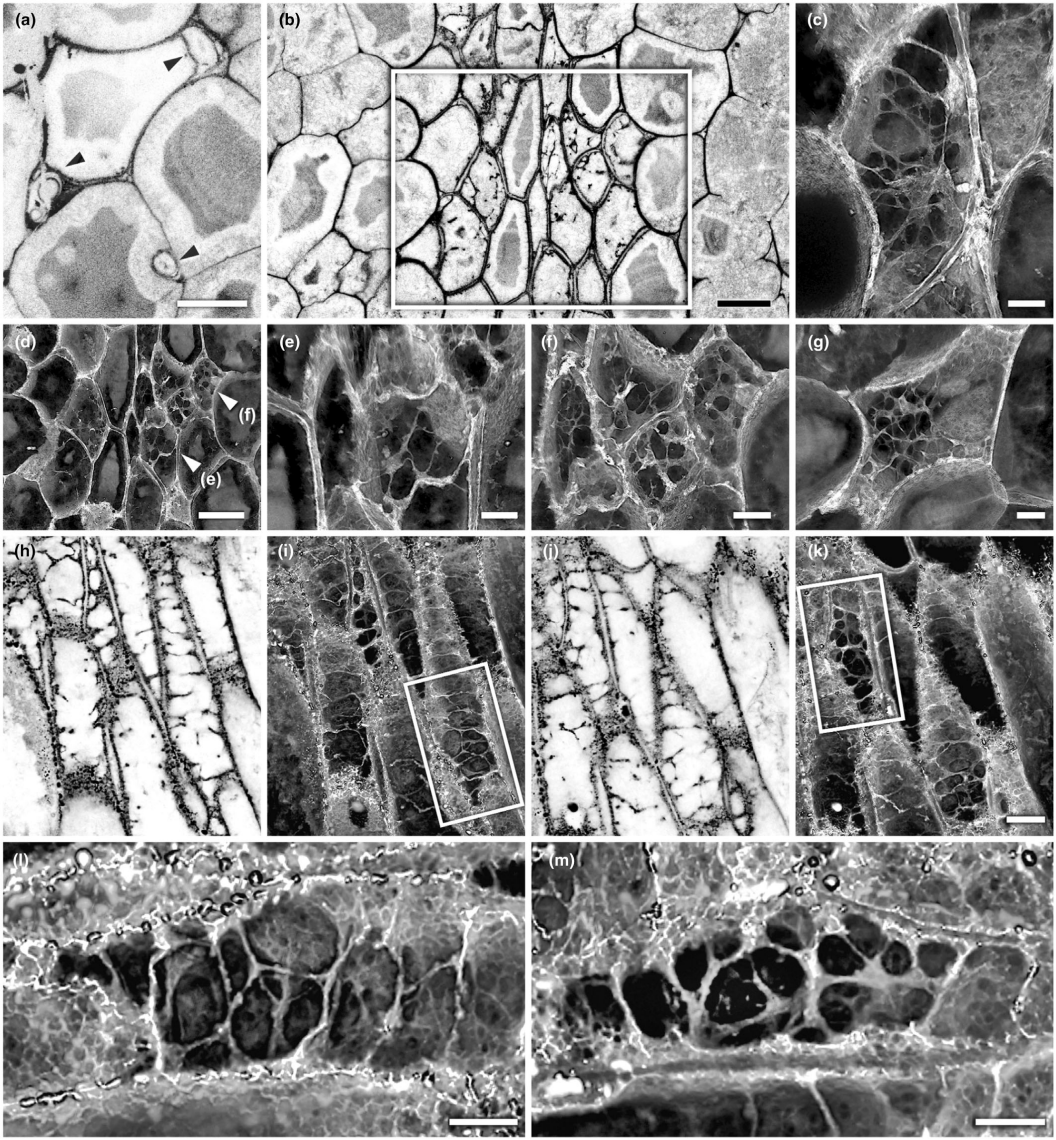
Confocal microscopy visualisations of conducting tissues and cortical cells in *Horneophyton lignieri*. (a–g) Oblique transverse section of cells in the rhizome centre. Details of Fig. 3(b) (NHMUK PI In 24697). (a, b) Single confocal optical sections. (a) Cortical cells with fungal hyphae in transverse section (arrowheads). (b) Conducting cells (inset) and neighbouring cortical cells. (c–g) Volume renderings showing a labyrinth of threads and sheets pervading the cell lumina of selected conducting cells from the inset in (b). Regions of interest in panels (e) and (f) are indicated by arrowheads in panel (d). See Supporting Information Fig. S2 for boxed regions corresponding to panels (b–g). (h–m) Longitudinal sections of conducting cells in the basal region of the aerial axis (NHMUK SC 3137). (h–k) Pairs of single confocal optical sections (h, j) and volume renderings (i, k) from the same fields of view at different depths. (l, m) Volume renderings detailing the labyrinthine structures within the lumina of conducting cells. Bars: (a, b, d) 40 μm; (c, e–g) 15 μm; (k) 20 μm (h–j same scale); (i, m) 10 μm.

**Fig. 6:**
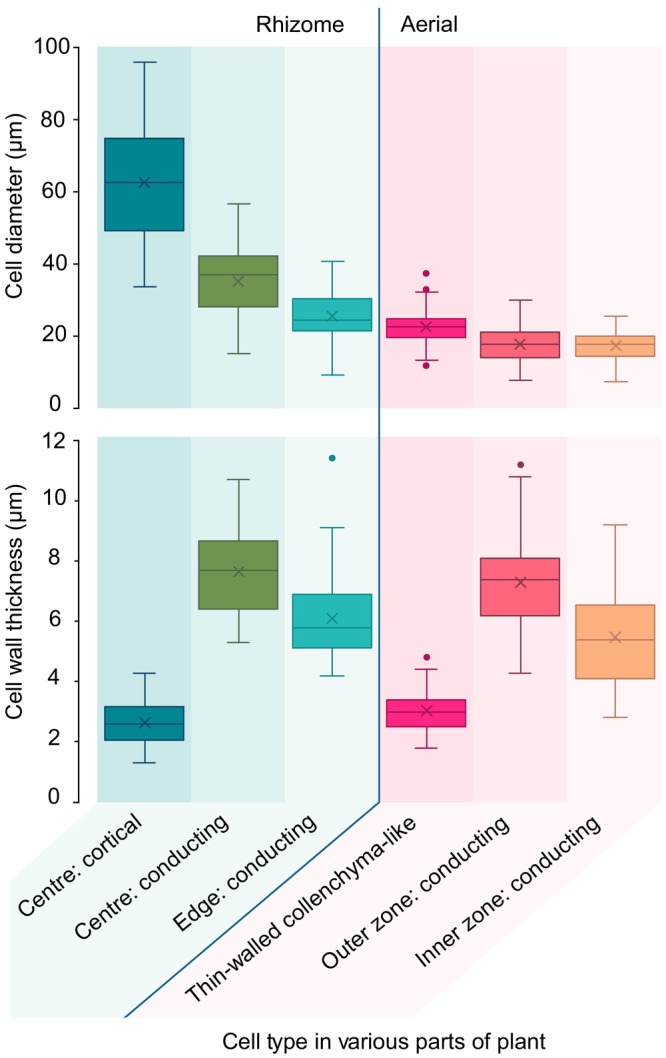
Box and whisker plots showing cell diameters (top) and cell wall thickness (bottom) for various tissue types in *Horneophyton lignieri*. (Top) Cortical cells in the rhizome are much larger in diameter than conducting cells. The diameter of conducting cells varies by position, being largest in the rhizome and basal regions of the aerial axes and narrowing in more distal regions of the plant. (Bottom) Cell walls of conducting cells are consistently thicker than the walls of the cortical cells and the collenchyma‐like cells. Each box represents the interquartile range (IQR), encompassing the middle 50% of measurements. The horizontal line within each box marks the median, and X indicates the mean. Whiskers extend to the smallest and largest values within 1.5 times the IQR from the quartiles, indicating the spread of typical data. Points plotted beyond the whiskers denote outliers – cells with dimensions significantly different from the rest of the sample.

A cluster of conducting cells, measuring *c*. 0.7 mm long and 0.3 mm wide, and their neighbouring cortical cells were imaged in detail (Figs 3b, 4a–d, 5a–g, S2). The section is oblique, causing cells in both tissues to appear slightly elongated. Comparing their major and minor axes shows that the conducting cells are at least 10% more elongate than the cortical cells (Fig. 4a,b). As the minor axis is less affected by the obliquity of the section, it provides a more accurate estimate of cell diameter. By this measure, the diameter of cortical cells is 44% greater than that of the conducting cells (62.5 μm compared with 35.3 μm average diameter), and the measured area of individual cortical cells is, on average, 60% larger. The cortical cells of the rhizome are therefore much larger in cross‐section than the adjacent conducting cells (Figs 4a,b, 5a,b, 6; Table S2).

Cortical cells have uniformly thin walls (average 2.6 μm) (Fig. 6; Table S4). Narrow intercellular spaces are occasionally visible, and in places, these spaces are colonised by fungal hyphae, which invaginate into neighbouring plant cells to form pouch‐like structures (Fig. 5a). Within the cell lumina, an outer zone of paler silica surrounds an inner zone of darker silica (Figs 4b, 5a,b). This indicates that there were two distinct phases of silicification: an initial phase of silica deposition focused on and adjacent to the cell walls and a later phase that infilled the remaining lumen cavities. Conducting cells have much thicker walls than cortical cells (average 7.6 μm) and have a more complex morphology. Typically, the walls between adjacent cells are tripartite, defined here as consisting of two dark, lumen‐facing layers divided by a paler intermediate layer (Fig. 4b). This reflects a difference in fluorescence that implies a difference in underlying chemistry. Within each cell, there is a highly interconnected labyrinth of irregularly shaped threads and sheets that pervades the lumen (Figs 4b–d, 5b–g). The threads are of variable size, often < 1 μm thick, with a wisp‐like quality, whereas the sheets are thin and highly variable in extent. Neither completely occludes the cell lumen. In places, the threads bear papillate outgrowths. This labyrinth is barely discernible using WLM, but it is clearly resolvable using CLSM (Figs 4c,d, 5c–g). Unlike the contact walls between neighbouring conducting cells, the walls between cortical parenchyma and conducting cells are single‐layered (Fig. 4b) and thicker (average 6.3 μm) than the cortical cell walls.

#### Conducting cells of the rhizome edge and base of the aerial axis

A second rhizome on slide NHMUK PI In 24697 was selected for analysis because it shows brown‐walled conducting cells near the base of an aerial axis (Fig. 2b). This rhizome appears to have a single lobe measuring 3 mm in width. The aerial axis is fragmented due to brittle fracture during a later stage of silicification. WLM shows relatively intact cortical tissues, conducting cells and fungal hyphae.

Conducting cells at the rhizome edge are more elongate than those at the rhizome centre (Figs 3c, 4e–h). Measurements of their minor axes indicate an average diameter of 25.8 μm, 27% narrower than the conducting cells of the rhizome centre (Fig. 6; Table S2). Cell walls are also *c*. 20% thinner (average 6.1 μm) (Fig. 6; Table S4). Like the conducting cells of the rhizome centre, adjacent cell walls are tripartite (Fig. 4f). Within each cell, irregularly shaped threads and sheets protrude into or extend across the lumen (Fig. 4f–h; S3a–d). In the peripheral cells, these form an interconnected labyrinth that pervades the lumen (Figs 4h, S3a). Moving inward, these structures take on a more thread‐like form of variable thickness, in places adhering to the cell wall, while in others extending across the lumen with no discernible regularity (Figs 4g, S3b,c). Similar features were observed in more longitudinally oriented cells at the base of the aerial axis, located directly above in the same section, and likewise on slide NHMUK SC 3137 at the transition between aerial axis and rhizome (Fig. 5h–m). It is the cells in this part of the conducting system that, under WLM, give the impression of faint tracheid‐like thickenings (Fig. 3d,e). Unlike the wall thickenings of tracheids, in addition to adhering to the wall in places, these structures also form interconnected threads that span the cell lumina (Figs 5h–m, S3h). Returning to the rhizome in NHMUK PI In 24697, the character of the wall changes towards the centre of the conducting tissues. Here, cells develop distinctive but highly irregular papillae that protrude into the lumen. Generally, the papillae do not extend across the lumen (Figs 4g,h, S3d).

Based on cell wall structure and position within the conducting strand, we distinguish two concentric zones within the conducting tissue of the rhizome edge and at the base of the aerial axis: zone 1, a central core of thick‐walled cells bearing papillate ingrowths; and zone 2, a peripheral band of thick‐walled cells with a labyrinth of threads or sheets that pervade the lumen. These zones are used hereafter to describe conducting strand tissues across both the rhizome and aerial axes.

#### Conducting cells of the aerial axes

**Fig. 7:**
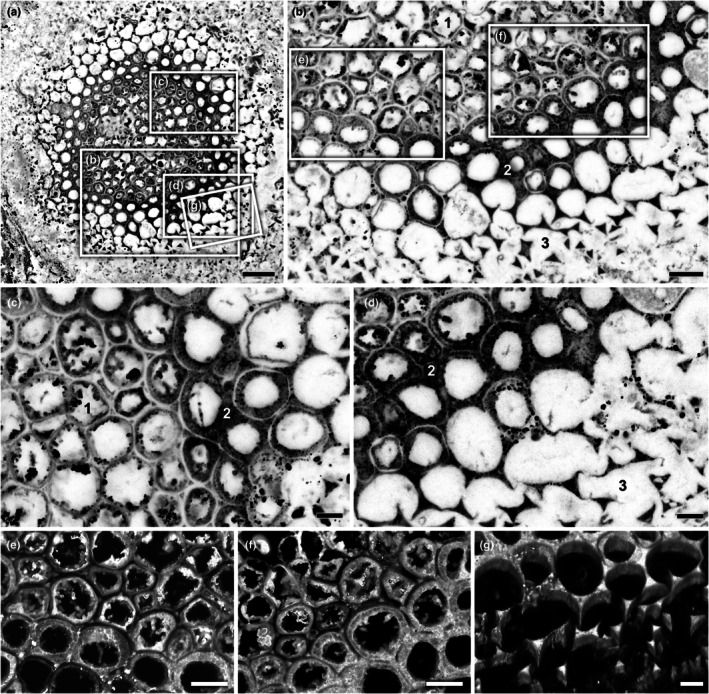
Confocal microscopy visualisations of transverse sections through conducting tissues and associated cells in the aerial axes of *Horneophyton lignieri*. Details of Fig. 3(a) (NHMUK OC 1938). (a) Whole conducting strand showing an outer ring of thin‐walled cells and an inner cylinder of thick‐walled cells corresponding to the phloem and xylem, respectively, of Kidston & Lang (1920). (b) Detail of conducting strand showing variation in cell wall thickness and structure across three distinct zones: zone 1, conducting cells with numerous papillate intrusions and thinner walls (previously interpreted as inner xylem); zone 2, conducting cells with a few papillate intrusions and uniformly thick walls (previously interpreted as outer xylem); and zone 3, thin‐walled collenchyma‐like cells with differential thickening at the corners (previously interpreted as phloem). (c) Close‐up of wall variation in conducting cells in zones 1 and 2. (d) Detail of the conducting cells of zone 2 and neighbouring thin‐walled collenchyma‐like cells of zone 3, which show corner thickenings. (e, f) Volume renderings of confocal image stacks illustrating the three cell types. (e) Conducting cells from the inner zone 1 showing papillate protrusions. (f) Conducting cells of zones 1 and 2. Papillate protrusions are fewer in the outer zone 2 cells, which also exhibit thicker walls. (g) Thin‐walled collenchyma‐like cells of zone 3 showing differential wall thickening at the corners. Bars: (a) 50 μm; (b) 20 μm; (c, d) 10 μm; (e, f) 15 μm; (g) 10 μm.

Aerial axes are less well preserved than rhizomes; generally, only the thick‐walled conducting tissues remain, often showing cell wall collapse or breakage. Under WLM, these cells appear orange‐brown, like the conducting tissues of the rhizome (Fig. 3). In the NHM slide collections, aerial axes are rarely observed in transverse section. However, on slide NHMUK OC 1938, a small cluster of axes is preserved in transverse or near‐transverse section, and one of these was imaged in detail (Figs 2c, 3a, S1b). The outer cortical tissues have decomposed, leading to the collapse of the axis perimeter and, in places, the ingress of spores from the surrounding sediment (Fig. 2c). The maximum diameter of the axis is 1.7 mm. The central vascular cylinder is relatively well‐preserved (Fig. 7). It is slightly subcircular in outline (400 × 370 μm in diameter) with an irregular, undulating margin (Fig. 7a), and comprises concentric zones of thick‐walled cells: a central core and a peripheral band, corresponding to zones 1 and 2, respectively, as defined in the rhizome (Fig. 7a,c). These zones are encircled by an additional, outer ring of thin‐walled cells, here designated zone 3 (Fig. 7a,d). Based on comparisons with Kidston & Lang (1920), zones 1 and 2 correspond to the inner and outer xylem, respectively, while zone 3 represents the phloem.

The cells of zone 3 generally have thin walls (average 3.0 μm thick) and wide lumina (average 22.8 μm in diameter) (Figs 6, 7a,b; Tables S2, S4). However, the walls exhibit subtle differential thickening. As noted by Kidston & Lang (1920), the wall at the junction where three cells meet is thicker, and as cells decompose, this is often the last part to break down, presenting as numerous small, triangular features (Figs. 7d,g, S4c,d). The intervening section of thinner (i.e. unthickened) wall can occasionally be seen preserved intact without apparent breakage, as in the upper left quadrant of the conducting strand (Fig. 7a). Within the thick‐walled conducting tissue of zones 1 and 2, two distinctive cell types are discernible. Zone 1 cells, forming the inner core, possess numerous papillate wall ingrowths (Figs 7c,e,f, S4). In a few places, these ingrowths are so obtrusive as to occlude the lumen. Zone 2, which encircles this core and is two to three cells thick, exhibits cells with thicker walls in which the papillate ingrowths are much reduced in number or absent. Like in the rhizome, the walls of adjacent cells are tripartite (Figs 7c, S4). The paler inner wall layer is more distinctive in the cells of zone 1 and sometimes absent in zone 2, where walls appear more uniformly thick. Although there is no significant difference in cell diameter between zones 1 and 2, which average 17.7 μm and 17.5 μm respectively (*P*‐value = 0.68 *t*‐test; Tables S2, S3), cell wall thickness differs significantly: zone 2 cells average 7.3 μm, compared with an average of 5.5 μm in zone 3 (*P*‐value < 0.05 *t*‐test; Tables S4, S5).

In longitudinal sections, only thick‐walled conducting cells were observed. WLM of an aerial axis on slide NHMUK PI In 24697 shows thick‐walled conducting cells but no plant tissues external to this, only a few fungal hyphae (Fig. 3f). Two subtly different wall textures are evident: a relatively uniform outer zone and a mottled inner zone. CLSM shows that these correspond to zones 2 and 1, respectively (Fig. S3e–g). The outer zone cells have uniformly thick walls with few or no papillae, whereas the inner zone cells have slightly thinner walls with numerous papillate ingrowths. Similar observations were made in other specimens. On slide NHMUK SC 3137, another feature noted by Kidston & Lang (1920) can be seen: it is common to observe repeated transverse breaks or interruptions within the cells of zone 1 (Fig. 3g), indicating that the inner zone of tissue is more susceptible to decomposition than the surrounding outer zone 2.

#### Overview of the conducting system

**Fig. 8:**
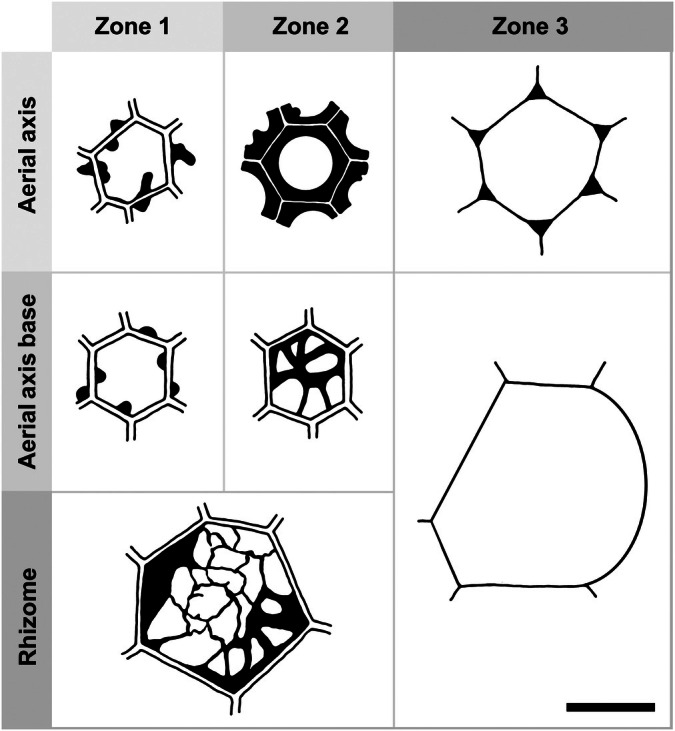
Diagrams of various cell types within and associated with the vascular system of *Horneophyton lignieri* in transverse section. In the aerial axis, the inner core (zone 1) consists of conducting cells with papillate wall ingrowths, while the surrounding band (zone 2) comprises thick‐walled conducting cells with few or no papillae and uniformly thick walls. The conducting strand (zones 1–2) is bordered externally by a ring of thin‐walled, collenchyma‐like cells (zone 3) with distinct corner thickenings. At the base of the aerial axis, zone 1 retains papillate ingrowths like those seen distally, whereas zone 2 conducting cells exhibit thinner cell walls adorned with labyrinthine wall structures. Zone 3 comprises larger, thin‐walled cortical cells that lack corner thickenings. In the rhizome, conducting cells in zones 1 and 2 are larger than those in the aerial axes and display complex labyrinthine wall architectures, while the cortical cells of zone 3 closely resemble those at the base of the aerial axis. Bar, 20 μm.

The conducting system is composed of cell types that change at different levels within the plant (i.e. rhizome, aerial axis; Figs 1b, 8), but which share some common features. A core of conducting cells with thick, tripartite walls extends continuously from the central region of the rhizome into the aerial axes. In the centre of the rhizome, these cells are comparatively short and wide; in the aerial axes, they become elongated and up to 50% narrower. In the centre of the rhizome, conducting cells are all of one type, characterised by a highly interconnected labyrinth of irregularly shaped threads and sheets that pervade the lumen. Toward the rhizome edge, the wall structure becomes zoned: peripheral cells (corresponding to zone 2) retain the labyrinthine architecture, while cells in the central core (zone 1) exhibit papillate wall ingrowths. This core of papillate‐walled cells persists into the aerial axes; however, the surrounding zone (zone 2) loses its internal labyrinthine structure and develops uniformly thick walls with few or no internal thickenings. In the rhizome, the core of thick‐walled conducting cells (zones 1 and 2) directly abuts the cortex, composed of significantly larger, thin‐walled cells. By contrast, in parts of the aerial axis, the conducting strand core (zones 1 and 2) is bordered by a narrow ring of elongate, thin‐walled cells with corner thickenings (corresponding to zone 3).

### Comparisons with tracheids and other types of conducting cells

#### The conducting cells of *Horneophyton lignieri* are not tracheids

Our findings reveal that the thick‐walled conducting cells in the core of the vascular system of *H. lignieri*, here termed H‐type cells, vary by position within the plant and differ markedly from the tracheids of vascular plants. CLSM reveals features undetectable (or barely discernible) under WLM, including the tripartite structure of adjacent cell walls, labyrinthine networks within cell lumina in the rhizome and basal aerial axes, papillate protrusions in central conducting strand cells, and a peripheral ring of uniformly thick‐walled cells with fewer or no papillae at higher levels of the upright axes.

By comparison, tracheids in vascular plants of this age possess wall thickenings with relatively large helical, annular or reticulate connections of a consistent thickness that protrude a short distance into the cell lumen. In the fossilised state, their walls have two layers: a highly mineralised inner layer and a thicker, coalified outer layer. These layers are thought to reflect original compositional differences. By contrast, the wall ingrowths of *H. lignieri* that can traverse the cell lumen to form labyrinthine networks are highly variable in thickness, and they are solid in section, indicating a consistent internal structure.

The insubstantial annular or helical thickenings previously resolved by WLM (e.g. Kidston & Lang, 1920; Kerp, 2018; Cascales‐Miñana *et al*., 2019) are here understood to be optical artefacts arising from overlapping labyrinthine structures on separate focal planes. Furthermore, the previously reported absence of wall thickenings in xylem cells at higher levels in the aerial axes, formerly attributed to pre‐fossilisation decomposition (Kidston & Lang, 1920; Edwards, 2004), is more plausibly explained by natural variation in wall morphology. At these levels, wall structures are smaller, papillate, and less discernible under WLM. Our analysis thus refutes the long‐standing hypothesis that the vascular system of *H. lignieri* is composed of tracheids with annular and helical wall thickenings.

Earlier studies reported a size gradient in the core of the vascular system, with central cells narrower than peripheral ones (Kidston & Lang, 1920; Edwards, 2004; Kerp, 2018), suggesting a developmental pattern of protoxylem encircled by metaxylem. However, like Eggert (1974), we observed no significant differences in cell size. Measurements of H‐type conducting cells in a well‐preserved transverse section showed only a minor, statistically insignificant increase in diameter from the centre to the periphery (Table S3). The main structural differences instead lie in wall morphology: central cells have thinner walls with numerous papillate protrusions, whereas peripheral cells possess uniformly thick walls. The thinner walls of central cells likely increase their susceptibility to decomposition and collapse, which could create the false impression of a smaller cell size. Apparent size differences reported in prior studies may thus reflect varying stages of cellular decomposition rather than true developmental gradients.

#### Conducting cells of *Horneophyton lignieri* resemble transfer cells

The wall ingrowths characterising H‐type cells in the *H. lignieri* conducting system strongly resemble the secondary wall ingrowths of transfer cells in both morphology and likely mode of development. Transfer cells are specialised versions of common plant cells found in various organs and tissues, including the vascular system (Gunning & Pate, 1969; Gunning, 1977; Talbot *et al*., 2002; Offler *et al*., 2003; Andriunas *et al*., 2013). Their defining feature is the presence of irregular secondary wall ingrowths. These ingrowths may take various forms: small and papillate, long and filiform, or branching and anastomosing to create a labyrinth of wall material extending across the cell lumen (Gunning & Pate, 1969).

The ingrowths in *H. lignieri* resemble the reticulate type described by Talbot *et al*. (2002), which begins with the emergence of small papillae from the underlying wall at seemingly random loci. As described by McCurdy *et al*. (2008), these papillae can branch to varying degrees of complexity and laterally fuse to form a reticulate, labyrinthine network. New papillae can then develop on the surface of the initial layer, with this process repeating to construct a multi‐layered, fenestrated labyrinth. In mature labyrinths, fenestrations may become progressively filled, producing diverse morphologies. Because these ingrowths originate from discrete loci of papillate wall deposition, their morphology and development differ fundamentally from the secondary wall thickenings of tracheary elements (Talbot *et al*., 2002; McCurdy *et al*., 2008; Offler & Patrick, 2020).

The resemblance to transfer cells also aligns with functional interpretations. In transfer cells, the extensive wall surface area created by ingrowths enhances active transport across the plasmalemma, facilitating bulk solute flow into or out of the cell (Gunning & Pate, 1974; Gunning, 1977). These structural adaptations enable high transport rates at physiological bottlenecks, making transfer cells essential for nutrient distribution. In the vascular systems of modern tracheophytes, they occur along the collection, transport and release pathways of the phloem (Andriunas *et al*., 2013), and they have also been documented in leptoid‐like conducting cells (i.e. deuters) in the leaves of the moss *Polytrichum commune* Hedw. (Scheirer, 1983). In *H. lignieri*, labyrinthine wall ingrowths are most pronounced in the cells of the rhizome and basal aerial axes, where they likely facilitated solute loading into the conducting system of the aerial axes. Transfer cells generally arise through *trans*‐differentiation, the de‐differentiation of the host cell followed by re‐differentiation, but in meristems, differentiation and transfer cell identity can occur concurrently (Andriunas *et al*., 2013). Their distribution in *H. lignieri* implies *trans*‐differentiation from food‐conducting cells within the aerial axes and perhaps from conducting parenchyma in the rhizome.

#### In the absence of xylem, can the presence of phloem be sustained?

The evidence for phloem in *H. lignieri* has always been circumstantial (Edwards, 1993; Kerp, 2018). Kidston & Lang (1920) based their interpretation on the position of the tissue in relation to the xylem and on its cellular characteristics. The thin‐walled cells identified as phloem are narrower and more elongate than cortical cells, suggesting that they functioned as sieve elements. However, the diagnostic sieve pores in their end walls were not documented – understandably, given the limitations of early 20^th^‐century microscopy. Kidston & Lang (1920) further noted that the cell corners stood out distinctly, possibly due to wall thickenings or small intercellular spaces. In a later study, Eggert (1974) recognised this feature as unique to *H. lignieri* among the Rhynie Chert plants and concluded that the cell walls were thicker at the corners, resembling collenchyma rather than phloem. In this context, a further observation made by Kidston & Lang (1920) has relevance: they highlighted the close morphological similarity and developmental continuity between the putative phloem cells and those forming the central, sterile columella tissue of the sporangium. These cells are continuous with the phloem‐like cells of the subtending axis, whereas H‐type conducting cells are absent from the columella, an observation confirmed by subsequent studies (Eggert, 1974; El‐Saadawy & Lacey, 1979).

Our analysis confirms that the walls of the putative phloem cells are differentially thickened, typically at the corners, and that these regions are more resistant to decomposition (Figs 7b,d,g, S4c,d). Collenchyma cells are elongate, contain living protoplasts, and possess nonlignified walls that are thickened in a uniform or varied manner. Of the histological types recognised in living plants by Leroux (2012), the cells of *H. lignieri* most closely resemble lacunar collenchyma, where only the wall sections adjacent to intercellular spaces are thickened. Unlike the rigid, lignified walls of sclerenchyma, the cell walls of collenchyma can grow and continue to thicken during and after elongation. In extant plants, collenchyma serves as the primary supporting tissue in growing organs (Leroux, 2012), and its presence in the aerial axes of *H. lignieri* suggests a similar structural role rather than a conductive one.

#### Comparisons with conducting systems preserved in fossil charcoal

Our understanding of vascular tissues in early land plants is primarily informed by fossils of Early Devonian age preserved in minerals such as pyrite, its oxidation products, and carbonates. Preservation in silicates, as seen in the Rhynie Chert, or in charcoal that formed during wildfire, is much rarer (Edwards, 2003). One of the most significant early fossil charcoal sites is in Shropshire, UK. Dating to the Lochkovian Stage of the Devonian, it is just a few million years older than the Rhynie Chert (Edwards *et al*., 2022). This site preserves a flora of tiny plants, but due to the brittle nature of charcoal, the fossils fracture into minute fragments, rendering it difficult to reconstruct their growth forms or determine their taxonomic affinities.

Charcoal‐preserved fossils are studied primarily using scanning electron microscopy (SEM), which lends itself to imaging external morphology. Internal features are more difficult to observe, but they can be glimpsed on fractured surfaces. These reveal elongate cells in the core of the axes, which are on average slightly narrower (typically < 13 μm in diameter) than the H‐type cells of *H. lignieri* (average diameter of 17.6 μm at higher levels of upright axes). Edwards *et al*. (2003) identified at least 14 types of conducting systems, distinguished by variations in cell wall architecture and combinations of cell types. Common characteristics include networks of strands of varying thickness. These strands often adhere to cell walls but can also traverse the lumen as simple filaments, irregular outgrowths, or plate‐like structures. In some instances, the underlying wall is obscured by smooth, globular outgrowths occurring singly, in chains or in clusters. These outgrowths are solid in section and may extend into rod‐shaped projections that branch and fuse within the lumen. Such diverse wall ingrowths strongly resemble features in the conducting tissues of *H. lignieri*, notably the interconnected labyrinth of irregular threads and sheets, and the papillate protrusions.

Edwards *et al*. (2003) were cautious in attributing function to the varied cell types that they documented in fossil charcoal. Initially, they concluded that these cells probably played dual roles in water conduction and structural support. However, in a subsequent study designed to investigate the effects of wildfire on cell morphology (Edwards & Axe, 2004), they showed that burning modern plant tissues could induce the formation of strands or meshwork structures that traverse the cell lumen. They concluded that some of the features observed in charcoal‐preserved fossils might therefore have formed postmortem. The labyrinthine wall structures in the conducting cells of *H. lignieri* could not have originated in this way, as its tissues were never subjected to wildfire.

Other features observed in fossil charcoal bear such a striking resemblance to structures in modern plant cells that they can be interpreted with greater confidence. A recurring feature of the conducting cells is a wall layer lining the lumen that bears numerous minute pores, each measuring on average 120 nm in diameter (Edwards *et al*., 2003). Similar micropores were documented in the cell wall of an early type of fossil tracheid (Kenrick *et al*., 1991), and they are widespread in the water‐ and food‐conducting cells of modern mosses and liverworts (Ligrone *et al*., 2000; Edwards *et al*., 2003, 2021a). In bryophytes, such pores develop from plasmodesmata, which is a plausible origin for those in the fossils. While we did not observe micropores in the conducting cell walls of *H. lignieri*, this likely reflects the resolution limits of the confocal system used in this study, which has a maximum optical resolution of 0.27 μm, insufficient to resolve features as small as 120 nm. While advanced CLSM systems may achieve higher resolution, the pores described in fossil charcoal thus fall below the detection threshold of our imaging setup (see table S1 in Long *et al*., 2024a). In principle, SEM could detect pores of this size in Rhynie Chert plants. However, SEM imaging of Rhynie fossils requires acid etching, but silicification renders cell walls quite fragile and they readily collapse, making good preparations difficult to obtain (Kenrick & Crane, 1991).

Food‐conducting cells and transfer cells have also recently been documented in fragments of fossil charcoal (Edwards *et al*., 2021a,b). The transfer cells are particularly striking, forming complex branched labyrinths that arise from multiple points on the cell walls. Transfer cells were documented in fossils with a thalloid morphology and inside the core of two axes, where they were associated with other cells likely involved in food conduction. The axes are very small, roughly 10% the width of a typical aerial axis of *H. lignieri*. Both the cells and their wall ingrowths in the charcoal fossils are generally narrower than those of *H. lignieri*, by as much as 50%. The dimensions of wall ingrowths in the thalloid fossils range from 180–470 nm and 55–110 nm in the axes, which fall within the ranges found in modern bryophytes (Edwards *et al*., 2021b). The dimensions of the labyrinth strands in *H. lignieri* are highly variable, especially where they merge, making consistent measurements difficult to achieve. Overall, their dimensions appear to exceed or fall within the upper end of the range observed in the charcoal fossils.

## Discussion

Our results demonstrate that the conducting system of *Horneophyton lignieri* consists of a solid core of thick‐walled cells that differ fundamentally from the tracheids in early vascular plants. These H‐type cells exhibit wall ingrowths that range from intricate labyrinths to simpler papillate protrusions, varying in both position and density within the conducting system. Their strong resemblance to transfer cells suggests a primary role in active solute transport, rather than passive water conduction as in tracheids. At higher levels in the plant axis, the peripheral cells of the conducting core have thicker walls with fewer or no papillate protrusions. This core of thick‐walled cells extends continuously from the central region of the rhizome through the aerial axes, terminating at the base of the sporangia.

The evidence for phloem in *H. lignieri* remains equivocal. The elongate cells encircling the core of H‐type conducting cells are restricted to the aerial axes of the plant, persisting into the central columella of the synangium. Their distinctively thickened corners make these parts of the wall more resilient as the tissue decomposes. These characteristics are not observed in the phloem of other Rhynie Chert plants. While thickenings of this type have been observed in the water‐conducting hydroids of a moss (Chavan *et al*., 2023), a water‐conducting function is unlikely here. In simple vascular systems, water‐conducting cells are invariably central and encircled by food‐conducting cells – opposite to the arrangement observed in *H. lignieri*. More compelling is the resemblance of these cells to lacunar collenchyma (Leroux, 2012). A plausible alternative is that they played a structural role as a support tissue during axis development.

Our findings build on those of Edwards *et al*. (2021b) who reported the first evidence of transfer cells in early land plant fossils. Two of their examples came from the cores of small axes preserved in charcoal, indicating that they were part of or associated with the conducting system. Due to the brittle nature of charcoal, the distribution and variation of transfer cells within these fossils cannot be determined. Nor is it possible to discern the affinities of the fossils, beyond making a general comparison with eophytes (*sensu* Edwards *et al*., 2021a). One feature may prove significant: *H. lignieri* is thought to be unique among the Rhynie flora in producing a branched or lobed synangium (Bhutta, 1973; Eggert, 1974; El‐Saadawy & Lacey, 1979). Intriguingly, one of the charcoal fossils described by Edwards *et al*. (Edwards *et al*., 2021b, Fig. 3g) also exhibits a cluster of bulbous structures suggestive of a similarly branched synangium.

Transfer cells are widespread in land plants, and they are occasionally found in other Archaeplastida and Fungi (Offler *et al*., 2003). Their presence across diverse organs and developmental contexts suggests an ancient and flexible genetic program (Offler *et al*., 2003; Andriunas *et al*., 2013). In *H. lignieri*, the extent to which ingrowths develop varies with position in the conducting strand. In modern plants, wall ingrowth development can also vary greatly both between and within cell types. Moreover, transfer cell differentiation is coordinated with organ development, occurring at specific locations across developmental windows (Offler *et al*., 2003). It seems likely, therefore, that in *H. lignieri*, variation in wall ingrowth development across different parts of the plant reflects local transport demands. In contrast to the wall thickenings of tracheids, this variation represents a calibrated physiological response rather than a fixed, predetermined pattern of cell wall deposition.

Our observations on *H. lignieri* lead us to propose a developmental and functional model summarised in our habit sketch (Fig. 1a). We suggest that the plant possessed a relatively large, persistent rhizome from which narrower, determinate, and short‐lived aerial axes arose. This interpretation is consistent with the relative dimensions of these organ systems and their differing states of preservation: rhizomes are consistently better preserved than aerial axes. The rhizome likely functioned as both a photosynthetic organ and a source of resources for developing aerial axes, whereas the primary role of the aerial axes was spore production and dispersal. Following sporogenesis, individual axes withered, and new spore‐producing axes developed sequentially from the rhizome. The aerial axes were probably also photosynthetic, sustaining their own limited growth but contributing little, if any, photosynthate to the rhizome. Solute transport was therefore short‐lived and largely unidirectional, from rhizome to aerial axis. While this model provides a coherent functional framework, it should be regarded as provisional pending further evidence on the organisation and physiology of the plant.

### Conclusions

*Horneophyton lignieri* holds significant evolutionary interest due to its Early Devonian age and unique characteristics. In their original description, Kidston & Lang (1920) noted that this ostensible tracheophyte possessed a key feature found only in some bryophytes, a sporangium with a well‐developed columella. The mature gametophyte was identified much later (Remy & Hass, 1991), and this also proved to be more bryophyte‐like in many respects. It is highly differentiated, with funnel‐shaped gametangiophores, glands, stomata, and conducting tissues (Kerp *et al*., 2004; Kerp, 2018). In their cladistic analysis, Kenrick & Crane (1997a,b) found *H. lignieri* to be a nonvascular plant that is the sister group to all other polysporangiophytes. Our findings are consistent with this hypothesis but also open the door to other potential relationships, including possible placement within the stem groups of bryophytes or embryophytes.

Crucially, *H. lignieri* lacks tracheids, severing a key link to the vascular plants, and it shows no clear evidence of water‐conducting cells. Its conducting system appears to consist entirely of solute‐conducting cells, supporting the hypothesis that such cells represent the ancestral condition in land plants. This finding is consistent with the hypothesis of a fundamental homology between food‐conducting tissues in both bryophytes and vascular plants (Xu *et al*., 2014; Donoghue *et al*., 2021; Edwards *et al*., 2021a).

Recent developments in molecular phylogenetics – from viewing bryophytes as paraphyletic to recognising them as a monophyletic group – challenge earlier assumptions about the nature of the most recent common ancestor of land plants (Puttick *et al*., 2018; Rensing, 2018; Harris *et al*., 2022). The morphological simplicity of plants like liverworts and hornworts may reflect derived states, evolutionary reduction and secondary loss rather than ancestral traits. Comparative genomics further supports this view: many gene families associated with vascular and stomatal functions are deeply conserved but have been lost in specific bryophyte lineages (de Vries & Archibald, 2018; Rensing, 2020; Donoghue *et al*., 2021; Bowles *et al*., 2022; Harris *et al*., 2022; Panstruga *et al*., 2025). Current understanding of plant phylogeny, together with new information from the fossil record and comparative genomics, therefore supports a deep origin and stepwise evolution of vascular tissues in land plants, with subsequent reduction and loss among some bryophytes (Woudenberg *et al*., 2022). Our observations on *H. lignieri* align with this interpretation and point to an ancestral conducting system composed of a single cell type capable of transporting both solutes and water – an early evolutionary stage in the origin of vascular tissues.

## Competing interests

None declared.

## Author contributions

PK and EJL contributed to the conceptualisation, analysis, investigation, project administration, and software (Avizo 3D, Nikon NIS Elements) use. EJL also handled data curation and CLSM methodology. PK carried out Fiji processing, statistical analysis, and wrote the original draft. EJL reviewed and edited the draft. PK supervised the work.

## Disclaimer

The New Phytologist Foundation remains neutral with regard to jurisdictional claims in maps and in any institutional affiliations.

## Supporting information


**Fig. S1** White light images of rhizomes and aerial axes of *Horneophyton lignieri*.
**Fig. S2** Rhizome centre conducting cells of *Horneophyton lignieri* imaged in white light indicating regions imaged in Fig. 5 using confocal microscopy.
**Fig. S3** Confocal microscopy data of conducting cells in rhizome and aerial axis of *Horneophyton lignieri*.
**Fig. S4** Confocal microscopy data of transverse sections of conducting cells and collenchyma‐like cells in the aerial axes of *Horneophyton lignieri*.
**Methods S1** Identification of specimens as *Horneophyton lignieri*.
**Table S1** Confocal laser scanning microscopy imaging parameters.
**Table S2** Cell diameters in the rhizome and aerial axes of *Horneophyton lignieri*.
**Table S3** Student's *t*‐test pairwise comparisons of cell diameters in the rhizome and aerial axes of *Horneophyton lignieri*.
**Table S4** Cell wall thickness in the rhizome and aerial axes of *Horneophyton lignieri*.
**Table S5** Student's *t*‐test pairwise comparisons of cell wall thickness of cells in the rhizome and aerial axes of *Horneophyton lignieri*.
Please note: Wiley is not responsible for the content or functionality of any Supporting Information supplied by the authors. Any queries (other than missing material) should be directed to the *New Phytologist* Central Office.

## Data Availability

Key CLSM datasets are available online at https://www.morphosource.org/projects/000699916.
