# Supplementary material for: Transfer cells in Horneophyton lignieri illuminate the origin of vascular tissues in land plants
Source: New Phytol. 2025 Dec 17;249(6):3149–64. doi: 10.1111/nph.70850 (PMC12917476; doi:10.1111/nph.70850)
Supplement: Supplementary file 1 — Fig. S1 White light images of rhizomes and aerial axes of Horneophyton lignieri. Fig. S2 Rhizome centre conducting cells of Horneophyton lignieri imaged in white light indicating regions imaged in Fig. 5 using confocal microscopy. Fig. S3 Confocal microscopy data of conducting cells in rhizome and aerial axis of Horneophyton lignieri. Fig. S4 Confocal microscopy data of transverse sections of conducting cells and collenchyma‐like cells in the aerial axes of Horneophyton lignieri. Methods S1 Identification of specimens as Horneophyton lignieri. Table S1 Confocal laser scanning microscopy imaging parameters. Table S2 Cell diameters in the rhizome and aerial axes of Horneophyton lignieri. Table S3 Student's t‐test pairwise comparisons of cell diameters in the rhizome and aerial axes of Horneophyton lignieri. Table S4 Cell wall thickness in the rhizome and aerial axes of Horneophyton lignieri. Table S5 Student's t‐test pairwise comparisons of cell wall thickness of cells in the rhizome and aerial axes of Horneophyton lignieri. Please note: Wiley is not responsible for the content or functionality of any Supporting Information supplied by the authors. Any queries (other than missing material) should be directed to the New Phytologist Central Office. [file NPH-249-3149-s001.docx]

## *New Phytologist* Supporting Information

Article title: Transfer cells in *Horneophyton lignieri* illuminate the origin of vascular tissues in land plants.

Authors: Paul Kenrick, Emma J. Long

Article acceptance date: 02 December 2025

The following Supporting Information is available for this article:

**Fig. S1** White light images of rhizomes and aerial axes of *Horneophyton lignieri*. (a) Section through rhizome showing two loci of brown-walled rhizome centre cells (BC1, BC2). Cells at the base of an aerial axis above BC2 (arrow) are shown at higher magnification in Fig. 5 (h–m) (NHMUK SC 3137). (b) Transverse section of nine aerial axes (i–ix) showing cellular preservation of central conducting tissues, one of which (i) is shown at higher magnification in Figs 2c, 3a, 7a–g, S4a–h (NHMUK OC 1938). Scale bars: (a) 1.0 mm; (b) 0.5 mm.


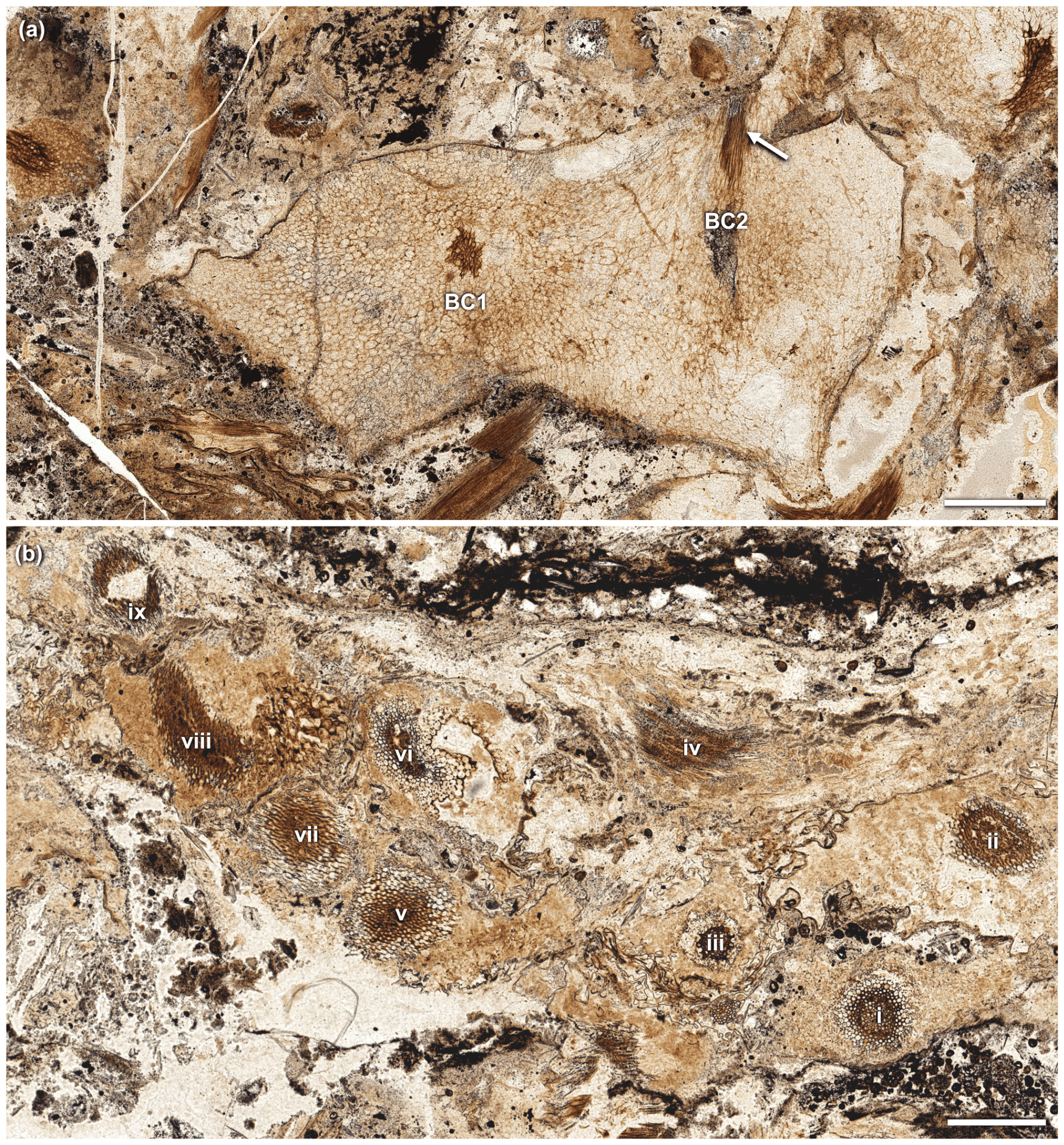


**Fig. S2** Rhizome centre conducting cells (central, with darker walls) and adjacent cortical cells (larger, with thinner walls) of *Horneophyton lignieri,* imaged using white light microscopy (NHMUK PI In 24697). Boxed regions corresponding to confocal image data in panels b–g of Fig. 5 in main text. Scale bar 100 µm.

**
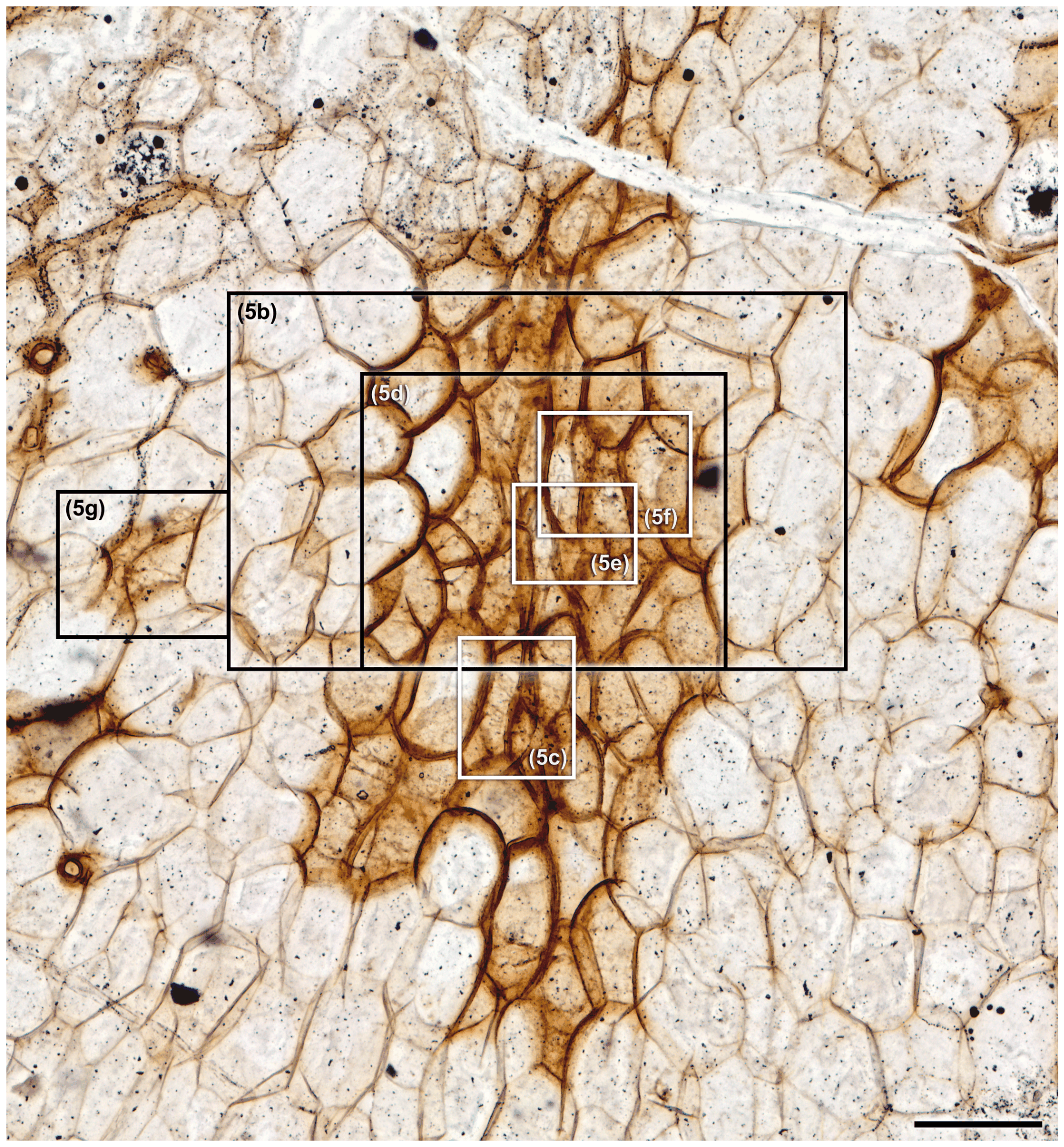
**

**Fig. S3** Confocal microscopy data of conducting cells in rhizome and aerial axis of *Horneophyton lignieri*. (a–d) Details of rhizome edge conducting cells shown at higher magnification and different orientations compared with Fig. 4g,h (NHMUK PI In 24697). (a–c) arrowheads indicate varied wall elements traversing lumina in peripheral cells. (d) arrows indicate peg-like thickenings of central cells (e, f) Pair of single confocal optical sections and corresponding volume renderings illustrating the greater abundance of peg-like wall thickenings in cells at the core of the conducting strand in higher levels of the aerial axis (NHMUK PI In 24697). (g) Detail from (f), tilted and shown at a higher magnification. (h) Volume rendering showing networks of thread-like wall thickenings within conducting cell lumina at base of aerial axis (NHMUK SC 3137). Scale bars: (a–d, g) 10 µm; (e, f, h) 20 µm.

**
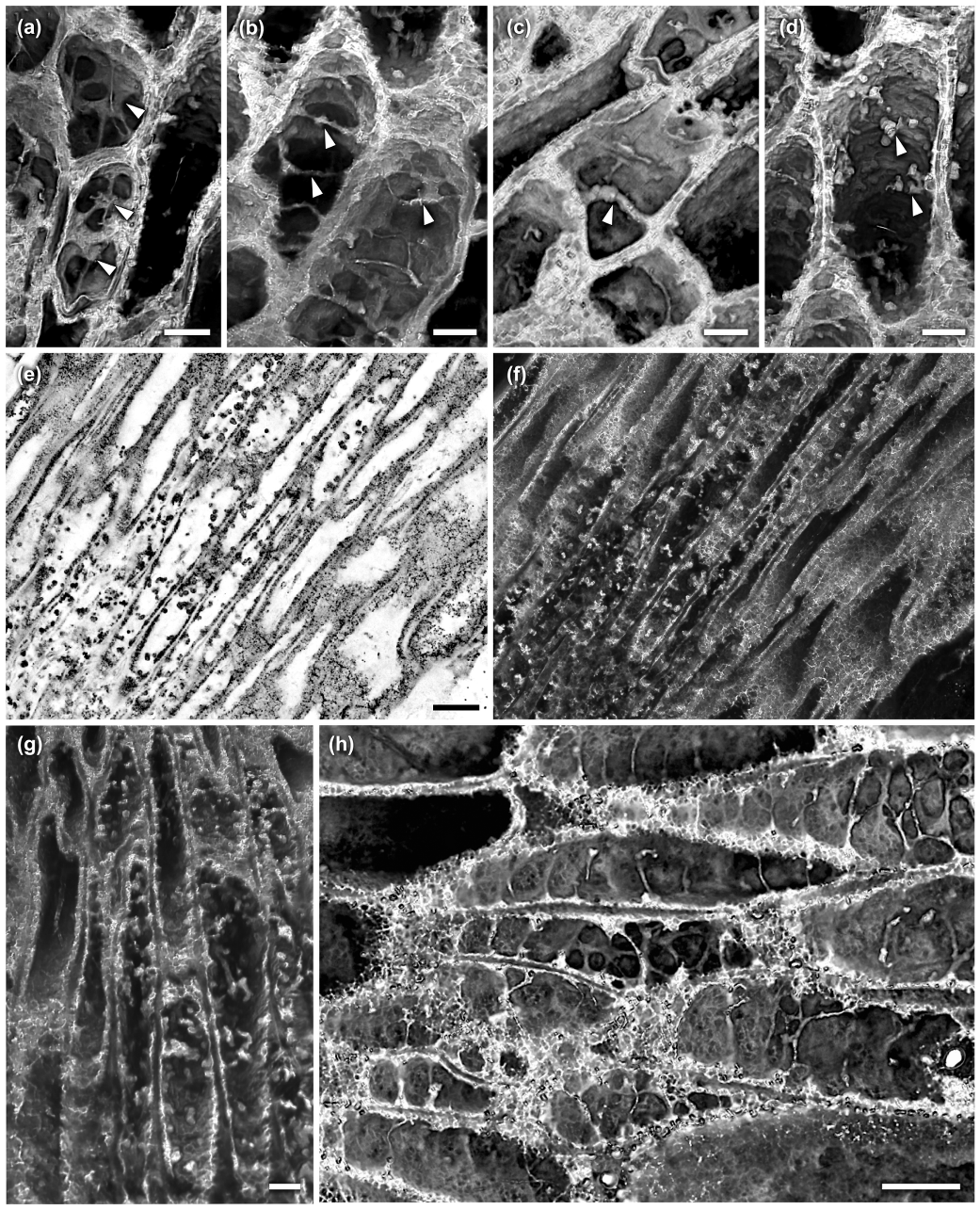
**

**Fig. S4** Confocal microscopy data of transverse sections of conducting cells and collenchyma-like cells in the aerial axes of *Horneophyton lignieri*. Pairs of single confocal optical sections (a, c, e, g) and corresponding volume renderings (b, d, f, h). Detail from Fig. 7a (NHMUK OC 1938). (a, b) Wall variation across the inner (1) and outer zones (2) of conducting cells. (c, d) Wall variation across the outer zone of conducting cells (2) and adjacent thin-walled collenchyma -like cells (3), which show differential thickening at the corners. (e–h) Higher magnification of papillate protrusions in conducting cells. Scale bars: (a–d) 20 µm; (e–h) 15 µm.


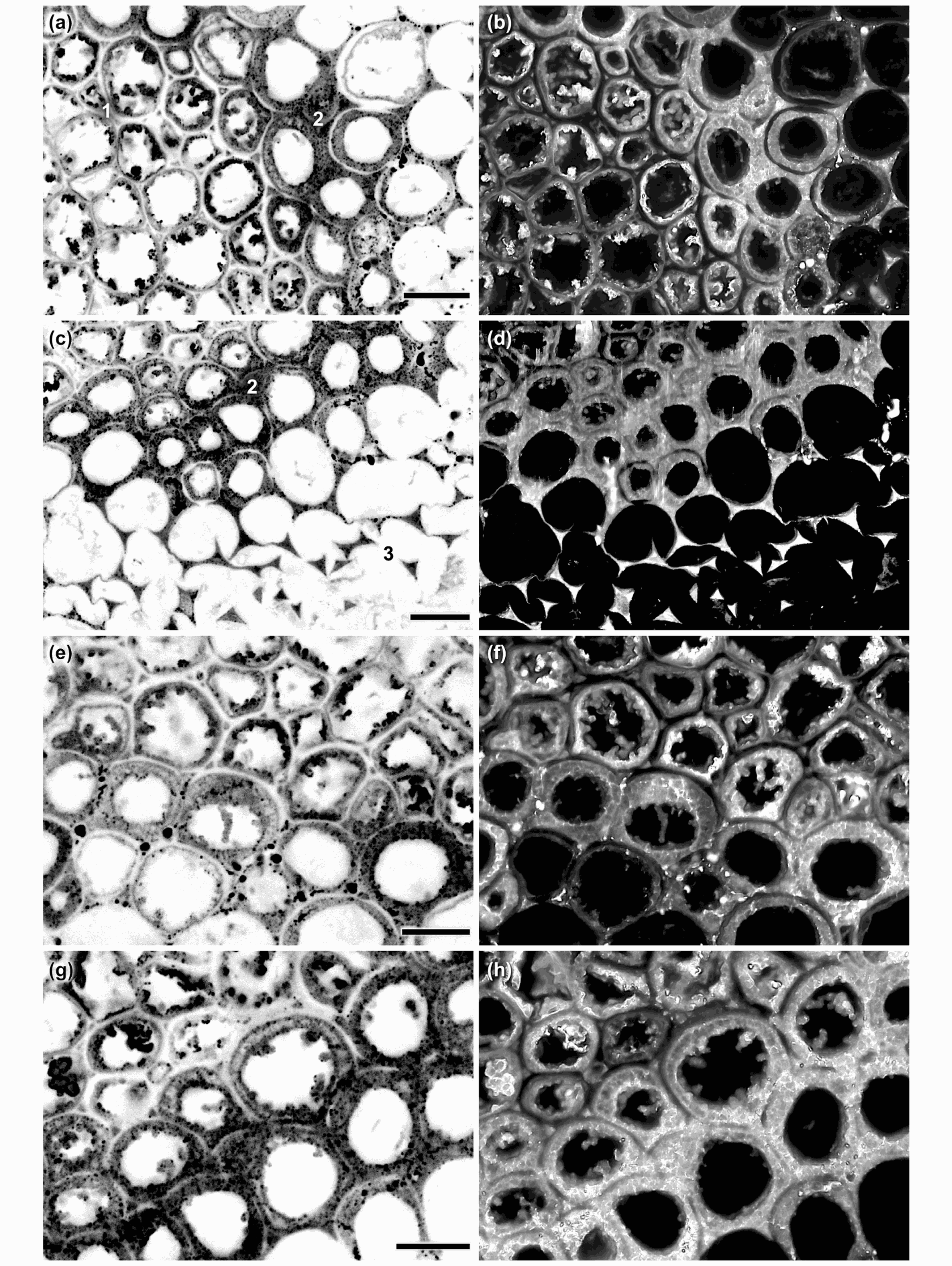


**Table S1** Confocal laser scanning microscopy (CLSM) imaging parameters of *Horneophyton lignieri*. All scans were conducted at wavelength 640.4 nm.

| Specimen | Figs | Objective lens | Digital zoom | XY pixel size (pixels/µm) | Z step size (µm) | No. Z steps | XY tile scan size (pixels) | Scan depth (µm) | CLSM pinhole (µm) | Line avg. | Pixel dwell time (µs) | XY scan area (tiles) | XY tile overlap (%) | MorphoSource doi |
| --- | --- | --- | --- | --- | --- | --- | --- | --- | --- | --- | --- | --- | --- | --- |
| NHMUK PI In 24697 | 4b–d, 5a–g | Plan Fluor 40x Oil DIC H N2 | X1 | 0.31 | 0.25 | 215 | 1024  x  1024 | 53.50 | 30.7 | 4X | 6.1 | 3 x 3 | 15 | [10.17602/M2/M757323](https://eur03.safelinks.protection.outlook.com/?url=https%3A%2F%2Fdoi.org%2F10.17602%2FM2%2FM757323&data=05%7C02%7Ce.long%40nhm.ac.uk%7C466d9e110f7d4643359208de22d280a9%7C73a29c014e78437fa0d4c8553e1960c1%7C1%7C0%7C638986484680178627%7CUnknown%7CTWFpbGZsb3d8eyJFbXB0eU1hcGkiOnRydWUsIlYiOiIwLjAuMDAwMCIsIlAiOiJXaW4zMiIsIkFOIjoiTWFpbCIsIldUIjoyfQ%3D%3D%7C0%7C%7C%7C&sdata=SwvphnQRI1E7elmHcvHJyr7oHEQJALglhYSrNyfYbAM%3D&reserved=0) |
|  | 4f–h, S3a–d | Plan Fluor 40x Oil DIC H N2 | X1 | 0.31 | 0.25 | 196 | 1024  x  1024 | 24.50 | 30.7 | 16X | 6.1 | 1 x 1 | n/a | [10.17602/M2/M757328](https://eur03.safelinks.protection.outlook.com/?url=https%3A%2F%2Fdoi.org%2F10.17602%2FM2%2FM757328&data=05%7C02%7Ce.long%40nhm.ac.uk%7C466d9e110f7d4643359208de22d280a9%7C73a29c014e78437fa0d4c8553e1960c1%7C1%7C0%7C638986484680211891%7CUnknown%7CTWFpbGZsb3d8eyJFbXB0eU1hcGkiOnRydWUsIlYiOiIwLjAuMDAwMCIsIlAiOiJXaW4zMiIsIkFOIjoiTWFpbCIsIldUIjoyfQ%3D%3D%7C0%7C%7C%7C&sdata=J0cDToHLs6NgTXAS%2FkvZ79GyEeTH3UWBha%2F8mgg6v40%3D&reserved=0) |
|  | S3e–g | Plan Fluor 40x Oil DIC H N2 | X1 | 0.31 | 0.25 | 178 | 1024  x  1024 | 44.25 | 30.7 | 16X | 13.7 | 1 x 1 | n/a | [10.17602/M2/M757319](https://eur03.safelinks.protection.outlook.com/?url=https%3A%2F%2Fdoi.org%2F10.17602%2FM2%2FM757319&data=05%7C02%7Ce.long%40nhm.ac.uk%7C466d9e110f7d4643359208de22d280a9%7C73a29c014e78437fa0d4c8553e1960c1%7C1%7C0%7C638986484680129169%7CUnknown%7CTWFpbGZsb3d8eyJFbXB0eU1hcGkiOnRydWUsIlYiOiIwLjAuMDAwMCIsIlAiOiJXaW4zMiIsIkFOIjoiTWFpbCIsIldUIjoyfQ%3D%3D%7C0%7C%7C%7C&sdata=Gb1vySkiFjrDgtXAxPGS4P6y%2F0iY1bPp7eRE8l6fVIY%3D&reserved=0) |
| NHMUK SC 3137 | 5h–m, S3h | Plan Fluor 40x Oil DIC H N2 | X1 | 0.31 | 0.25 | 255 | 1024  x  1024 | 62.50 | 30.7 | 16X | 6.1 | 1 x 1 | n/a | [10.17602/M2/M757342](https://eur03.safelinks.protection.outlook.com/?url=https%3A%2F%2Fdoi.org%2F10.17602%2FM2%2FM757342&data=05%7C02%7Ce.long%40nhm.ac.uk%7C466d9e110f7d4643359208de22d280a9%7C73a29c014e78437fa0d4c8553e1960c1%7C1%7C0%7C638986484680291998%7CUnknown%7CTWFpbGZsb3d8eyJFbXB0eU1hcGkiOnRydWUsIlYiOiIwLjAuMDAwMCIsIlAiOiJXaW4zMiIsIkFOIjoiTWFpbCIsIldUIjoyfQ%3D%3D%7C0%7C%7C%7C&sdata=KxwaG2g2JwHebKW7l%2FsKNxwksJ7X2wM6c%2BXX5czhrIg%3D&reserved=0) |
| NHMUK OC 1938 | 7a | Plan Fluor 20x Ph1 DLL | X1.26 | 0.48 | 2.65 | 17 | 1024  x  1024 | 42.40 | 40.9 | 16X | 13.7 | 1 x 1 | n/a | [10.17602/M2/M699953](https://doi.org/10.17602/M2/M699953) |
|  | 7b–g, S4 | Plan Fluor 40x Oil DIC H N2 | X1 | 0.08 | 0.25 | 65 | 4096  x  4096 | 16.00 | 30.7 | 16X | 1.5 | 1 x 1 | n/a | [10.17602/M2/M757336](https://eur03.safelinks.protection.outlook.com/?url=https%3A%2F%2Fdoi.org%2F10.17602%2FM2%2FM757336&data=05%7C02%7Ce.long%40nhm.ac.uk%7C466d9e110f7d4643359208de22d280a9%7C73a29c014e78437fa0d4c8553e1960c1%7C1%7C0%7C638986484680250628%7CUnknown%7CTWFpbGZsb3d8eyJFbXB0eU1hcGkiOnRydWUsIlYiOiIwLjAuMDAwMCIsIlAiOiJXaW4zMiIsIkFOIjoiTWFpbCIsIldUIjoyfQ%3D%3D%7C0%7C%7C%7C&sdata=IoOauU2Jvs5AWCpKJejkbljLDONfMQstbuNnrXB86kI%3D&reserved=0) |

**Table S2** Cell diameters (µm) in the rhizome and aerial axes of *Horneophyton lignieri*.

|  | NHMUK PI In 24697* | | | NHMUK OC 1938** | | |
| --- | --- | --- | --- | --- | --- | --- |
|  | Rhizome Centre  Conducting  (Zone 1+2) | Rhizome Centre Cortical  (Zone 3) | Rhizome Edge Conducting  (Zone 1+2) | Aerial Conducting Inner  (Zone 1) | Aerial Conducting Outer  (Zone 2) | Aerial  Thin-walled  Collenchyma-like  (Zone 3) |
| Average | 35.3 | 62.5 | 25.8 | 17.5 | 17.7 | 22.8 |
| Median | 37.2 | 62.6 | 24.7 | 17.9 | 17.8 | 22.6 |
| Variance S | 107.3 | 283.9 | 38.0 | 15.7 | 24.5 | 24.9 |
| Skew | -0.1 | 0.1 | 0.0 | -0.3 | 0.1 | 0.4 |
| Max | 56.6 | 95.9 | 40.7 | 25.8 | 30.3 | 37.5 |
| Min | 15.3 | 33.7 | 9.4 | 7.5 | 7.9 | 12.0 |
| Number | 30 | 42 | 48 | 92 | 92 | 60 |

*Measurements are based on the minor axis where sections are oblique; **averages of major and minor axes are reported where sections are transverse.

**Table S3** Student’s *t*-test pairwise comparisons of cell diameters in the rhizome and aerial axes of *Horneophyton lignieri.*

|  | NHMUK PI In 24697 | | NHMUK OC 1938 | | |
| --- | --- | --- | --- | --- | --- |
|  | Rhizome Centre Cortical  (Zone 3) | Rhizome Edge Conducting  (Zone 1+2) | Aerial Conducting Inner  (Zone 1) | Aerial Conducting Outer  (Zone 2) | Aerial  Thin-Walled Collenchyma-like  (Zone 3) |
|  |  |  |  |  |  |
| Rhizome Centre Conducting (Zone 1+2) | 2.95E-12 | 4.61E-05 | 1.79E-10 | 2.19E-10 | 3.58E-07 |
| Rhizome Centre Cortical (Zone 3) |  | 3.28E-18 | 8.16E-21 | 6.62E-21 | 4.01E-19 |
| Rhizome Edge Conducting  (Zone 1+2) |  |  | 3.16E-12 | 1.99E-11 | 9.04E-03 |
| Aerial Conducting Inner (Zone 1) |  |  |  | **6.83E-01** | 2.20E-10 |
| Aerial Conducting Outer (Zone 2) |  |  |  |  | 8.36E-09 |

Tests were two-tailed, assuming two-sample unequal variance. Value in bold indicates result not significant (*p*  ≥ 5.0E-02). Excluding comparisons between the inner and outer zones of aerial conducting cells, all comparisons show statistically significant differences in cell diameter (*p* < 0.05).

**Table S4** Cell wall thickness (µm) in the rhizome and aerial axes of *Horneophyton lignieri.*

|  | NHMUK PI In 24697 | | | | | NHMUK OC 1938 | | | | |  |
| --- | --- | --- | --- | --- | --- | --- | --- | --- | --- | --- | --- |
|  | Rhizome Centre Conducting  (Zone 1+2) | Rhizome Centre Cortical  (Zone 3) | | Rhizome Edge Conducting  (Zone 1+2) | | Aerial Conducting Inner  (Zone 1) | Aerial Conducting Outer  (Zone 2) | | Aerial  Thin-Walled Collenchyma-like  (Zone 3) | |  |
| Average | 7.6 | 2.6 | | 6.1 | | 5.5 | 7.3 | | 3.0 | |  |
| Median | 7.7 | 2.6 | | 5.8 | | 5.4 | 7.4 | | 3.0 | |  |
| Variance S | 2.1 | 0.5 | | 2.1 | | 2.8 | 2.4 | | 0.5 | |  |
| Skew | 0.2 | 0.3 | | 1.4 | | 0.3 | 0.5 | | 0.4 | |  |
| Max | 10.7 | 4.3 | | 11.4 | | 9.2 | 11.2 | | 4.8 | |  |
| Min | 5.3 | 1.3 | | 4.2 | | 2.8 | 4.3 | | 1.8 | |  |
| Number | 20 | 20 | | 48 | | 50 | 50 | | 44 | |  |
|  |  | |  | |  |  | |  | |  | |

**Table S5** Student’s *t*-test pairwise comparisons of cell wall thickness of cells in the rhizome and aerial axes of *Horneophyton lignieri.*

|  | NHMUK PI In 24697 | | | NHMUK OC 1938 | | |
| --- | --- | --- | --- | --- | --- | --- |
|  | | Rhizome Centre Cortical (Zone 3) | Rhizome Edge Conducting  (Zone 1+2) | Aerial Conducting Inner  (Zone 1) | Aerial Conducting Outer  (Zone 2) | Aerial  Thin-Walled Collenchyma-like  (Zone 3) |
| Rhizome Centre Conducting (Zone 1+2) | | 3.74E-14 | 3.07E-04 | 3.00E-06 | 2.91E-02 | 1.52E-12 |
| Rhizome Centre Cortical  (Zone 3) | |  | 4.79E-10 | 2.41E-07 | 1.05E-12 | 4.07E-04 |
| Rhizome Edge Conducting (Zone 1+2) | |  |  | 4.75E-02 | 1.61E-04 | 1.48E-20 |
| Aerial Conducting Inner  (Zone 1) | |  |  |  | 1.72E-07 | 6.84E-14 |
| Aerial Conducting Outer  (Zone 2) | |  |  |  |  | 4.33E-27 |

Tests were two-tailed, assuming two-sample unequal variance. All comparisons show statistically significant differences in cell wall thickness (*p* < 0.05).

**Methods S1** Identification of specimens as *Horneophyton lignieri*.

All specimens examined were preserved in petrographic thin sections prepared from blocks of Rhynie Chert by the professional slide maker Walter Hemingway (1859–1947). The slides are labelled *Hornea lignieri*, a homotypic synonym of *Horneophyton lignieri* (Barghoorn & Darrah, 1938). In the slides we examined, rhizomes and aerial axes of *H. lignieri* are abundant, occurring almost to the exclusion of other plants. This observation is consistent with that of Kidston and Lang (1920), who reported that remains of this species occur in considerable quantities, locally forming nearly pure stands.

In the thin sections, only short lengths of the axes can be followed. The identity of individual axes was therefore confirmed anatomically by comparison with the original descriptions of organs and tissues by Kidston and Lang (1920), supplemented by subsequent accounts (Eggert, 1974; Edwards, 2004; Kerp, 2018).

Rhizomes and aerial axes of *H. lignieri* are readily distinguished from those of other Rhynie Chert plants. Cellular preservation in the rhizomes is typically more complete than in the aerial axes (Fig. 2a,b; Supporting Information Fig. S1a). The rhizome is substantially larger than the axes it bears, parenchymatous, and lacks a distinct vascular system. The conducting strand of the aerial axis, dark brown in colour, terminates blindly within the rhizome parenchyma, forming a cluster of approximately isodiametric, dark-walled cells (Fig. 3b). Rhizomes also bear unicellular rhizoids.

In our material, aerial axes are more common and markedly narrower than rhizomes. They are clearly distinct from the axes of other Rhynie Chert plants. Several aerial axes were observed attached to rhizomes, although they more commonly occur as short, isolated fragments. Typically, only the cells of the conducting strand are preserved (Fig. 3). In longitudinal sections near the base of the axis, these cells show faint wall thickenings. Under white light microscopy (Fig. 3d,e), the thickenings are notably less regular and robust than those of the tracheids in other Rhynie Chert plants. At higher levels, thickenings are not visible under white light (Fig. 3f). The conducting strand frequently exhibits horizontal breaks, a feature not seen in other Rhynie Chert plants (Fig. 3g). One slide contained nine aerial axes in transverse section (Fig. S1b). In this view, the cells surrounding the conducting tissue show corner thickenings, a feature unique to *H. lignieri* (Fig. 7b,d,g).

In summary, the materials we examined and figure here correspond precisely with the detailed anatomical descriptions of *H. lignieri* given in the original account by Kidston and Lang (1920).

Full literature citations are provided in the main text.
